# Genome Assembly Revealed MdZAT5 Coordinates Anthocyanin Biosynthesis in Apple Fruit Peel and Flesh by Interacting With MdHY5

**DOI:** 10.1111/pbi.70434

**Published:** 2025-11-03

**Authors:** Mengnan Zhao, Mosen Zhang, Weilei Sun, Shiyao Duan, Jie Zhang, Tingting Song, Yaoxin Wang, Yuncong Yao, Ji Tian

**Affiliations:** ^1^ Beijing Advanced Innovation Center for Tree Breeding by Molecular Design Beijing University of Agriculture Beijing China; ^2^ Plant Science and Technology College Beijing University of Agriculture Beijing China; ^3^ College of Biological Sciences and Technology Beijing Forestry University Beijing China; ^4^ Smartgenomics Technology Institute Tianjin China

**Keywords:** anthocyanin, apple, flesh, fruit, light, MeJA, peel

## Abstract

Anthocyanins are plant pigments that contribute to fruit coloration and nutritional quality, yet the coordinated regulation of their accumulation in both peel and flesh remains elusive. Here, we present a haplotype‐resolved genome of *Malus* cv. ‘Royalty’, a model cultivar with consistently red peel and flesh. A zinc‐finger transcription factor, MdZAT5, was identified as a candidate regulator of anthocyanin biosynthesis, with two copies located on chromosomes 3 and 11 in each haplotype, as revealed by a well‐assembled genome and transcriptomic profiling. Promoter and expression analyzes indicated that *MdZAT5‐3G* acts downstream of light and MeJA signalling pathways. Transgenic analysis showed that MdZAT5‐3G significantly promotes anthocyanin accumulation and upregulates anthocyanin biosynthesis genes such as *MdCHS*, *MdCHI*, and *MdF3H*, whereas its knockdown leads to reduced pigmentation. In contrast, MdZAT5‐11G lacks regulatory function in both peel and flesh. Downstream binding assays confirmed that MdZAT5‐3G directly binds to the promoters of anthocyanin biosynthetic genes, and biochemical assays revealed that its interaction with MdHY5 enhances promoter binding and transcriptional activation.

## 1 Introduction

Anthocyanins are soluble plant pigments that confer a range of colours to plant tissues (Boss et al. 1996; Chen et al. 2019; Honda et al. 2005; Vaknin et al. 2005; Meng et al. 2023). Among these colours, red is particularly prominent and appealing in fruits, and is closely related to anthocyanin accumulation (Wang et al. 2017). Anthocyanins play multiple roles in enhancing environmental stress tolerance, resistance to pathogens, attracting pollinators, and facilitating seed dispersal (Li et al. 2022). Anthocyanins also bring numerous benefits to human health, playing an important role in the prevention of cancer, cardiovascular and cerebrovascular diseases, heart disease, diabetes, and degenerative conditions (Ayvaz et al. 2022; Ijinu et al. 2023).

The anthocyanin biosynthetic pathway in plants has gradually been revealed (Konczak and Zhang 2004). Anthocyanins are recognized to be synthesized through the phenylalanine pathway. This biosynthetic process is divided into early and late stages, and is conducted by early biosynthetic genes chalcone synthase (*CHS*), chalcone isomerase (*CHI*), flavanone3‐hydroxylase (*F3H*), and late biosynthetic genes dihydroflavonol 4‐reductase (*DFR*), anthocyanidin synthase/leucoanthocyanidin dioxygenase (*ANS/LDOX*), and UDP‐glucose: flavonoid 3‐*O* glucosyltransferase (*UFGT*) (Tanaka et al. 2008; Winkel‐Shirley 2001).

The anthocyanin biosynthesis genes were generally regulated at the transcriptional level by transcription factors (TFs). Studies have demonstrated that multiple TFs can participate in coordinating fruit peel coloration. In apple, MYB TFs play an important role in the regulation of anthocyanin biosynthesis pathways (Hichri et al. 2011). For example, *MdMYB1/A* are responsible for controlling anthocyanin biosynthesis in apple fruit peel by activating the expression of *MdUFGT*, *MdDFR* or *MdANS*, respectively (Ban et al. 2007). Meanwhile, *MdMYB9*, *MdMYB11*, *MdMYB12*, *MdMYB22* have been identified as positive regulators (An et al. 2012; Wang et al. 2017). Additionally, the basic leucine zipper (bZIP) transcription factor HY5 is known to be induced by light and Methyl‐jasmonic acid (MeJA), and MdHY5 may be involved in anthocyanin biosynthesis by directly activating *MdMYB10* (An et al. 2017; Prasad et al. 2012). *MdNAC52* (*NAM*, *ATAF1/2* and *CUC2*), whose gene transcript levels increased during apple coloration, could interact with the promoters of *MdMYB9* and *MdMYB11* to regulate anthocyanin biosynthesis (Sun et al. 2019). Recently, our studies have shown that ETHYLENE RESPONSE FACTOR 109 (ERF109) promotes coloration by binding directly to anthocyanin‐related gene promoters involved in light‐induced anthocyanin biosynthesis (Ma et al. 2021).

Meanwhile, many TFs have been identified that participate in apple flesh coloration. MdMYB10 (*R*_*6*_: *MdMYB10*) is constitutively expressed in the whole fruit via its direct binding to its own enhancer promoter, forming an autoregulatory loop that activates *MdMYB10* expression, ultimately leading to the development of red‐fleshed apples (Espley et al. 2007). The expression of *MYB10* is significantly correlated with anthocyanin accumulation in type I red‐fleshed apples (Espley et al. 2007). While MYB110a, a paralog of MYB10, is associated with type II red‐flesh phenotype in apple (Chagné et al. 2013). MdNAC1 acts as a positive regulator of anthocyanin synthesis in red‐fleshed apples by interacting with MdbZIP23 to form a transcriptional complex that significantly increases the transcription of *MdMYB10* and *MdUFGT* (Liu et al. 2023). MdWRKY10 interacted with MdTTG1 to join the MBW complex, regulating *MYB10* expression for anthocyanin synthesis in apple flesh (Wang et al. 2024). Together, these findings highlight the complexity of the transcriptional regulatory network. However, the synergistic regulatory mechanism underlying anthocyanin synthesis in apple peel and flesh remain to be fully elucidated.

Zinc finger transcription factors regulate several developmental and cellular processes in plants, including RNA binding, flowering time, plant defence, and stress response (Kiełbowicz‐Matuk 2012; Putterill et al. 1995; Yu et al. 2006). Recent studies have also shown that zinc finger proteins have the potential to regulate anthocyanin accumulation. In *Arabidopsis thaliana* , the expression of *ZINC FINGER of Arabidopsis thaliana* 6 (*ZAT6*) positively affects anthocyanin and total flavonoid concentrations by activating the expressions of *TT5*, *TT7*, *TT3*, *TT18*, *MYB12*, and *MYB111* through binding to their promoters with TACAAT elements of these genes (Shi et al. 2018). Similarly, in apple, a B‐box transcription factor gene, MdBBX20, which belongs to the zinc finger protein family, was characterized and identified to promote anthocyanin biosynthesis under UV‐B conditions (Fang et al. 2019). However, the role of zinc finger protein in anthocyanin accumulation in apple remains unclear.

Continued researches showed that anthocyanin regulators may perform function by forming protein complexes. *MdMYB1/A/10* interacted with two distinct bHLH proteins from apple, MdbHLH3 and MdbHLH33, to regulate fruit coloration (Ban et al. 2007; Takos et al. 2006; Espley et al. 2007). MdWRKY40 promotes wounding‐induced anthocyanin biosynthesis in apple and is identified as an MdMYB1‐interacting protein, enhancing the binding of MdMYB1 to its target genes in response to wounding (An, Wang, et al. 2019). MdERF38 has been proved to be involved in drought stress‐induced anthocyanin biosynthesis by interacting with MdMYB1, and facilitating the binding of MdMYB1 to downstream target genes (An, Wang, Espley, et al. 2020). MdMYB308L interactes with MdbHLH33 and enhances its binding to the promoters of *MdCBF2* and *MdDFR*, and participate in cold induced anthocyanin biosynthesis (An, Wang, Zhang et al. 2020). Meanwhile, MdBBX20 has been shown to interact with MdHY5 and that this interaction was required to significantly enhance the promoter activity of *MdMYB1*, to promote anthocyanin biosynthesis (Fang et al. 2019). Therefore, we deduced that zinc finger proteins may modulate fruit coloration by interacting with other regulatory proteins.

Apple ( *Malus domestica* Borkh.) is one of the most widely produced and economically important fruit crops in temperate regions (Duan et al. 2017; Upadhyay and Gupta 2024). Red coloration of apple fruit peel and flesh is an important determinant of consumer preference and marketability. Although many studies have been conducted on anthocyanin accumulation in apple fruit, metabolic pathways and underlying mechanisms of fruit peel and flesh coloration development still require further investigation. In this study, we attempt to obtain a TF that coordinate regulates anthocyanin biosynthesis in apple fruit peel and flesh. *Malus* crabapple cultivar ‘Royalty’ has purple‐red peel and flesh, with the colour covering the whole fruit from the beginning of fruit setting to maturation (Tian et al. 2015, 2017), making it an ideal material to study fruit coloration. Here, we report for the haplotype‐resolved genome sequences of ever‐red fruit cultivar ‘Royalty’. Using a well‐assembled genome sequence and the following transcriptome, a candidate anthocyanin regulator zinc finger TF, designated MdZAT5, was identified in anthocyanin correlated co‐expression modules. Genomic mapping identified two MdZAT5 copies located on chromosomes 3 and 11 in each haplotype. Biochemical and molecular assays demonstrated that MdZAT5‐3G promotes anthocyanin biosynthesis in apple peel and flesh by interacting with MdHY5 and enhancing the transcriptional activity of anthocyanin biosynthesis genes in response to light and MeJA induction. Our research reveals a potential regulating mechanism of anthocyanin accumulation in red apple fruit, which will help provide a basis for genetic improvement of anthocyanin accumulation in apple.

## 2 Results

### 2.1 Chromosome‐Scale Genome Assembly and Gene Annotations

**FIGURE 1:**
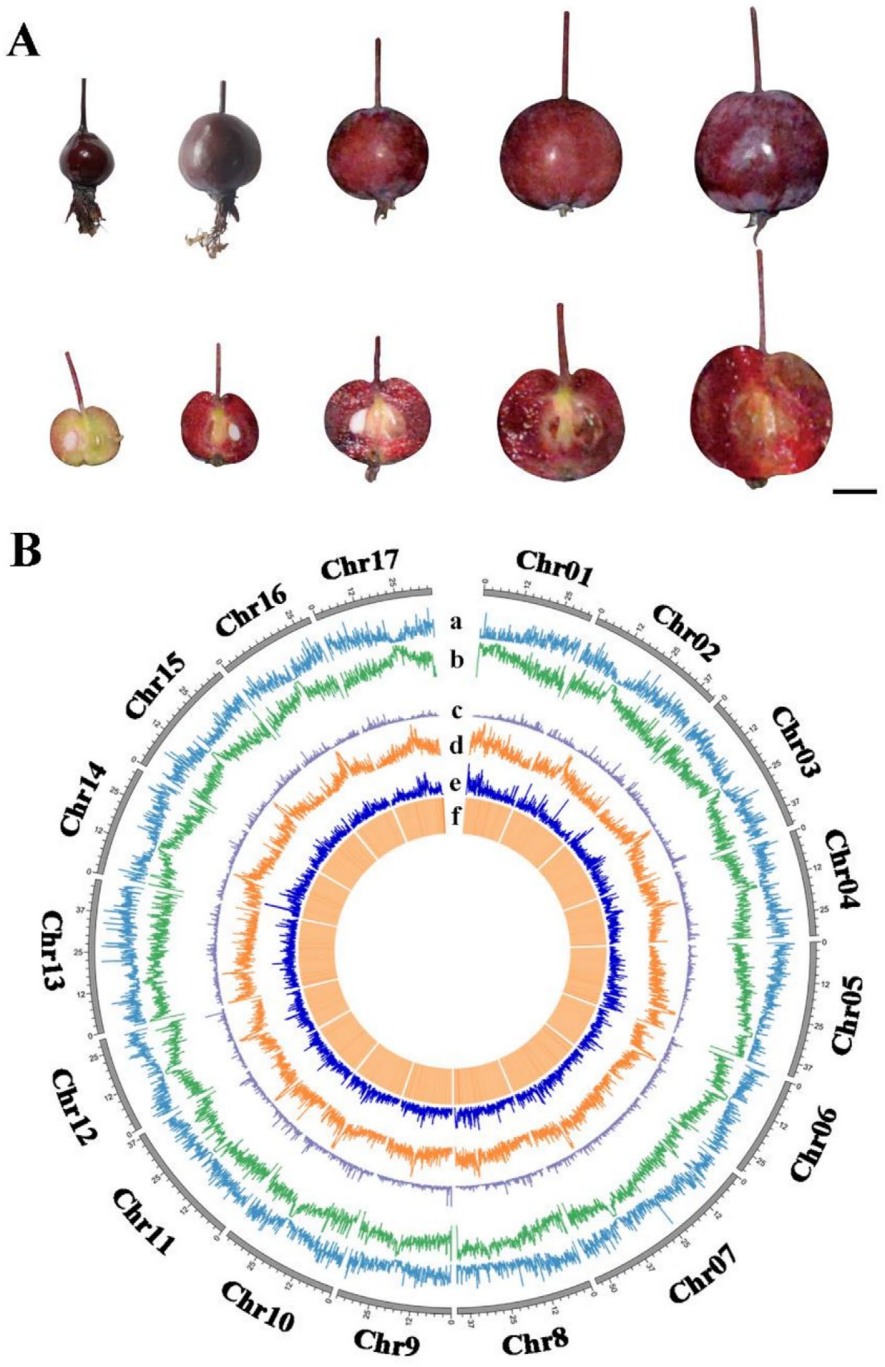
High‐quality genome assembly of the *Malus* crabapple ‘Royalty’. (A) Fruit phenotypes of ‘Royalty’ fruit during different developmental stages. (B) Genomic features of ‘Royalty’ genome. Tracks from outer to inner: (a) gene density, (b) transposable element (TE) density, (c) DNA transposons density, (d) Gypsy retrotransposons density, (e) Copia retrotransposons density, and (f) GC contents.

To construct a high‐quality genome of *Malus* crabapple ‘Royalty’ (Figure 1A), PacBio sequences (71.17 Gb, 101.89× sequence coverage) were generated to assemble the genome into contigs, yielding a draft assembly of 803 contigs with a total length of 675.82 Mb and contig N50 of 36.50 Mb mega base‐pairs (Tables 1 and S1). After polishing and quality improvement, an improved assembly with a scaffold N50 of 36.53 Mb was obtained (Table 1). Finally, Hi‐C interaction datasets (173.84 Gb, 248.88 × sequence coverage) were used to construct the genome into super‐scaffolds, and 95.12% of the assembled contigs were anchored into 17 chromosomes (Figure 1B, Tables 1, S2 and S3).

**TABLE 1:** Assembly and annotation features of ‘Royalty’ genomes.

Accession	*Malus* crabapple ‘Royalty’
Assembly	
Total length of scaffolds	675.82 Mb
Total number of scaffolds	787
Scaffold N50	36.53 Mb
Total length of contigs	675.82 Mb
Total number of contigs	802
Contig N50	36.50 Mb
Average contig length	842 749 bp
Sequences anchored to chromosome	95.12%
Complete BUSCOs	98.9%
LTR assembly index (LAI)	18.28
Annotation	
Percentage of repeat sequences	56.72%
Number of genes	46 507

Furthermore, 94.35% of the ultra‐conserved core eukaryotic genes based on Core Eukaryotic Genes Mapping Approach (CEGMA) analysis, and 98.9% of the single‐copy orthologs based on the Benchmarking Universal Single‐Copy Orthologs (BUSCO) analysis, could be completely detected in the assembly, further confirming the continuity and quality of the assembled genome (Tables S4 and S5) (Parra et al. 2007; Seppey et al. 2019). Meanwhile, the consensus quality value (QV) of the ‘Royalty’ genome was 47.68, and Completeness was 78.18%, indicating the high accuracy of our assembly (Table S6) (Rhie et al. 2020). In addition, by mapping short Illumina reads to the genome assembly, we obtained a mapping rate of 99.11%, a genome coverage of 99.91%, and an SNP rate of 0.81%, which further demonstrates the high quality of the assembled genome (Table S7).

We annotated the genome by incorporating de novo gene prediction, homology comparison, and transcriptome‐based annotation, resulting in 46 507 protein‐coding genes in the ‘Royalty’ genome (Tables 1 and S8). The numbers of annotated genes in ‘Royalty’ are close to that in the ‘Hanfu’ ( *Malus domestica* ) genome which has 44 677 genes (Zhang et al. 2019). Then, the predicted protein‐coding genes were annotated via BLAST searches against the nonredundant (NR), Swiss‐Prot, Kyoto Encyclopedia of genes and genomes (KEGG), InterPro, Protein families database (Pfam) and gene ontology (GO) database, and 94.8% of predicted protein coding genes could be annotated in these database (Table S9). In the GO analysis, 10 280 (22.10%), 3473 (7.47%) and 15 678 (33.71%) annotated protein‐coding genes were assigned to the GO slim terms biological process (BP), molecular function (MF) and cellular component (CC), respectively (Table S9). The set of predicted noncoding genes included 7723 ribosomal RNAs (rRNAs), 3981 transfer RNAs (tRNAs), 1090 microRNAs (miRNAs), 805 small nuclear RNA (snRNA) (Table S10). Taken together, these results indicated the high completeness and accuracy of our ‘Royalty’ genome.

### 2.2 Assessment of Haplotype‐Resolved Assembly

**FIGURE 2:**
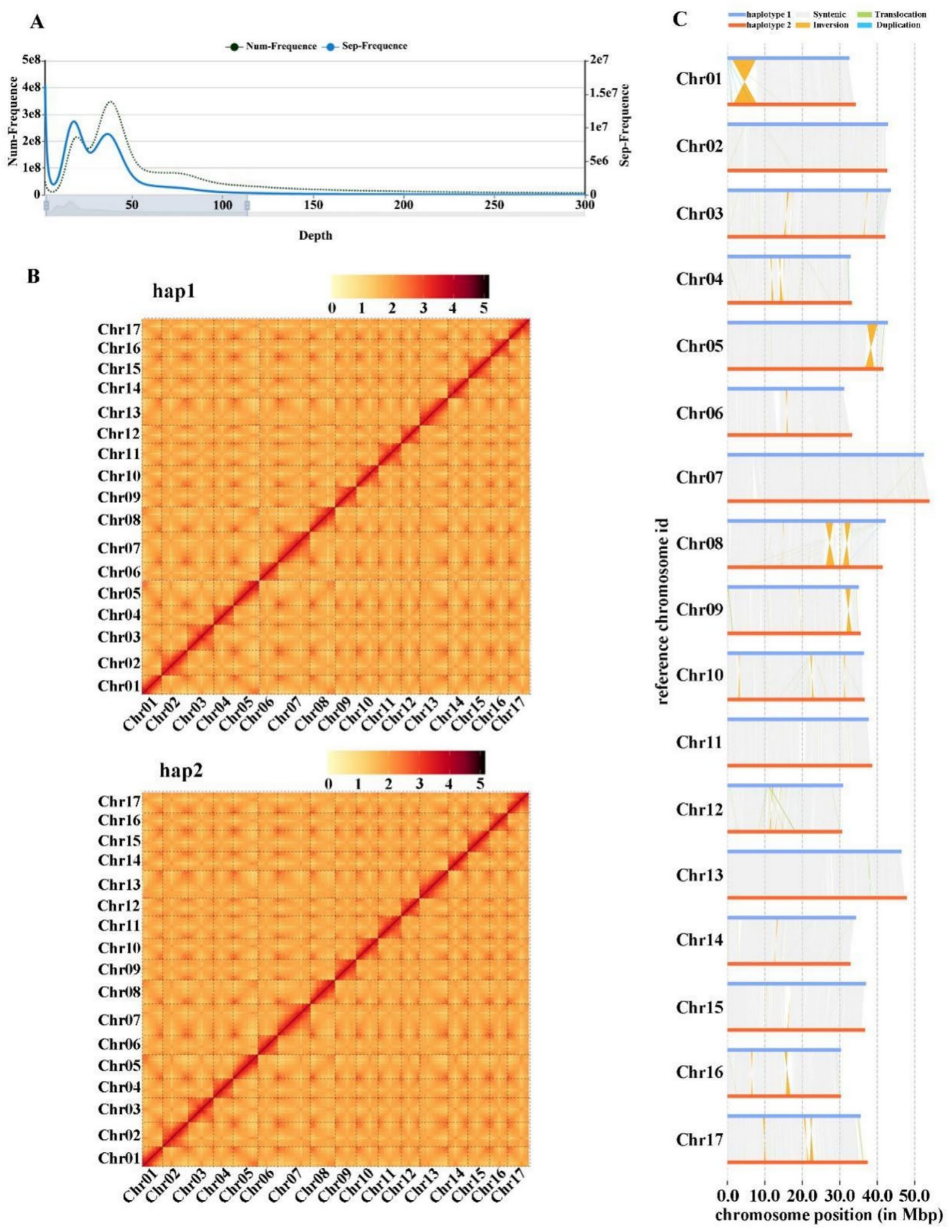
Overview of haplotype‐resolved genome analysis of *Malus* ‘Royalty’. (A) Evaluation of ‘Royalty’ genome size by *K‐mer* analysis. (B) Hi‐C interaction heatmap showing chromatin contact intensity among the 17 chromosomes. (C) Whole‐genome alignment between haplotype 1 (hap1) and haplotype 2 (hap2), indicating structural variations.

Haplotype‐resolved genome assembly was conducted using Hifiasm, resulting in two distinct contig sequences designated as hap1 (Royalty_hap1) and hap2 (Royalty_hap2). To further scaffold the assemblies, Hi‐C data were aligned using HiCUP, and the resulting interaction matrices were used for clustering, ordering, and orientation with ALLHIC. This process successfully anchored 636.43 Mb and 641.75 Mb of hap1 and hap2 sequences, respectively, onto 17 chromosomes. The chromosome anchoring rates were 95.44% for hap1 and 98.37% for hap2, with individual chromosomal lengths ranging from 29 829 781 bp to 53 941 138 bp (Table S11). Additionally, *K*‐mer analysis (*k* = 17) estimated the genome heterozygosity of ‘Royalty’ at 1.36%, indicative of a highly heterozygous diploid genome (Figure 2A).

Hi‐C interaction heatmap analysis demonstrated strong signal enrichment along the diagonal, representing adjacent genomic regions, while signal intensities were markedly reduced in the off‐diagonal regions, corresponding to non‐adjacent sequences. The clear separation between these regions, along with the absence of anomalous interaction signals outside the diagonal, confirmed the high accuracy of chromosome anchoring (Figure 2B).

The final Hi‐C‐assisted assemblies yielded genome sizes of 666.85 Mb for hap1 and 652.41 Mb for hap2. The scaffold N50 values were 36.53 Mb (hap1) and 36.98 Mb (hap2), while the contig N50 values reached 33.61 Mb and 35.72 Mb, respectively. Moreover, BUSCO analysis revealed high completeness scores of 98.7% for hap1 and 99.0% for hap2, further indicating that a high‐quality, haplotype‐resolved assembly of the ‘Royalty’ genome was successfully achieved (Table 2).

**TABLE 2:** Assembly and annotation features of ‘haplotype 1’ and ‘haplotype 2’.

Accession	Haplotype 1	Haplotype 2
Assembly		
Total length of scaffolds	666.85 Mb	652.41 Mb
Total number of scaffolds	756	211
Scaffold N50	36.53 Mb	36.98 Mb
Total length of contigs	666.84 Mb	652.40 Mb
Total number of contigs	862	263
Contig N50	33.61 Mb	35.72 Mb
Average contig length	773 596	2 480 609
Sequences anchored to chromosome	95.44%	98.37%
Complete BUSCOs	98.70%	99.00%
LTR assembly index (LAI)	17.53	19.34
Annotation		
Percentage of repeat sequences (%)	56.41	55.44
Number of genes	45 696	44 622

Genomic variation is a major contributor to genetic diversity and adaptive evolution, and may also play a critical role in speciation through recombination (Weischenfeldt et al. 2013). Structural variation refers to genomic alterations with a wide size range, including inversions, translocations, and duplications (or deletions). In this study, 293 major collinear blocks were identified between the two haplotypes through synteny analysis. Furthermore, a total of 176 042 structural variations (SVs) were detected between the two haplotypes using SyRI v1.5. Among these SVs, we identified 6877 deletions (DEL, 3.91%), 1719 duplications (DUP, 0.98%), 16 099 syntenic inversions (SYN, 9.14%), 17 898 non‐syntenic inversions (NOTAL, 10.17%), 5896 translocations (TRANS, 3.35%), and 18 238 homology‐directed repair events (HDR, 10.36%) (Table 3, Figure 2C).

**TABLE 3:** Distribution of chromosomal structural variation types.

Type	Number	Rate (%)
SYN (syntenic inversion)	16 099	9.14
SYNAL (syntenic inversion and DEL)	63 112	35.85
TRANS (translocation)	5896	3.35
TRANSAL (translocation and DEL)	6319	3.59
NOTAL (non‐synthetic inversion)	17 898	10.17
DUP (duplication)	1719	0.98
DUPAL (duplication and DEL)	1764	1.00
DEL (deletion)	6877	3.91
INVTR (inverted transposon)	5325	3.02
INVTRAL (inverted transposon and DEL)	5517	3.13
HDR (homology‐directed repair)	18 238	10.36
INS (insertion)	7338	4.17
CPL (complex structural variant)	8316	4.72
CPG (complex inversion)	8134	4.62
INVAL (insertion and inversion)	2415	1.37
INV (inversion)	200	0.11
TDM (traditional mobile element)	875	0.50

### 2.3 Annotation of ‘Royalty’ Haplotypes

Gene structures were predicted de novo using the software programs Augustus, GlimmerHMM, Geneid, and Genscan. Following prediction, homologous annotation was performed. For genes filtered out during the generation of the final gene set (Genefilter), each was individually assessed for functional annotation and transcript evidence support. Genes possessing functional annotation or exhibiting an expression level > 1 RPKM in at least one transcriptome sample were retained. The annotation process yielded a total of 45 696 and 44 622 protein‐coding genes for the hap1 and hap2 assemblies, respectively (Table 4). Subsequent alignment of the predicted protein sequences against the NR, Swiss‐Prot, KEGG, and InterPro databases revealed that 43 425 genes in hap1 and 42 411 genes in hap2 were functionally annotated (Table S12). These gene counts are comparable to the 47 563 and 48 655 protein‐coding genes annotated in the published ‘Honeycrisp’ haplotype genomes (Khan et al. 2022). These results were consistent with previously reported annotations in the ‘Honeycrisp’ haplotype genome, which contain 47 563 and 48 655 protein‐coding genes, respectively (Khan et al. 2022). The comparable gene numbers and high annotation rates between the current and published genomes underscore the reliability and completeness of the gene annotation in this study.

**TABLE 4:** Statistical analysis of gene structure annotation results of ‘Royalty’ haplotypes genome.

	Number	Average gene length (bp)	Average CDS length (bp)	Average number of exons per gene	Average exon length (bp)	Average intron length (bp)
hap1	45 696	3480.23	1229.19	4.79	256.39	593.27
hap2	44 622	3628.72	1242.18	4.92	252.3	608.27

### 2.4 Identification of MdZAT5 as a Potential Anthocyanin Regulator

Next, we sought to identify the specific transcription factors involved in regulating anthocyanin accumulation in the peel and flesh of ‘Royalty’ fruit using RNA‐seq analysis. A total of 657 differentially expressed genes (DEGs) were identified between the second and first stages of peel development, including 164 up‐regulated and 493 down‐regulated genes. Between the third and second stages, 4050 DEGs were detected, with 1455 up‐regulated and 2595 down‐regulated. From the fourth to the third stage, 5530 DEGs were identified, including 1934 up‐regulated and 3596 down‐regulated genes. Notably, 8916 DEGs were observed between the final and previous stages of peel development, of which 3180 were up‐regulated and 5736 were down‐regulated (Figure S1). In addition, anthocyanin content was measured in the peel and flesh of ‘Royalty’ fruit at five stages of development. The results demonstrated that cyanidin 3‐*O*‐glucoside is the predominant anthocyanin in ‘Royalty’ fruit, and its content gradually increased during fruit development in both peel and flesh (Figure S2).

**FIGURE 3:**
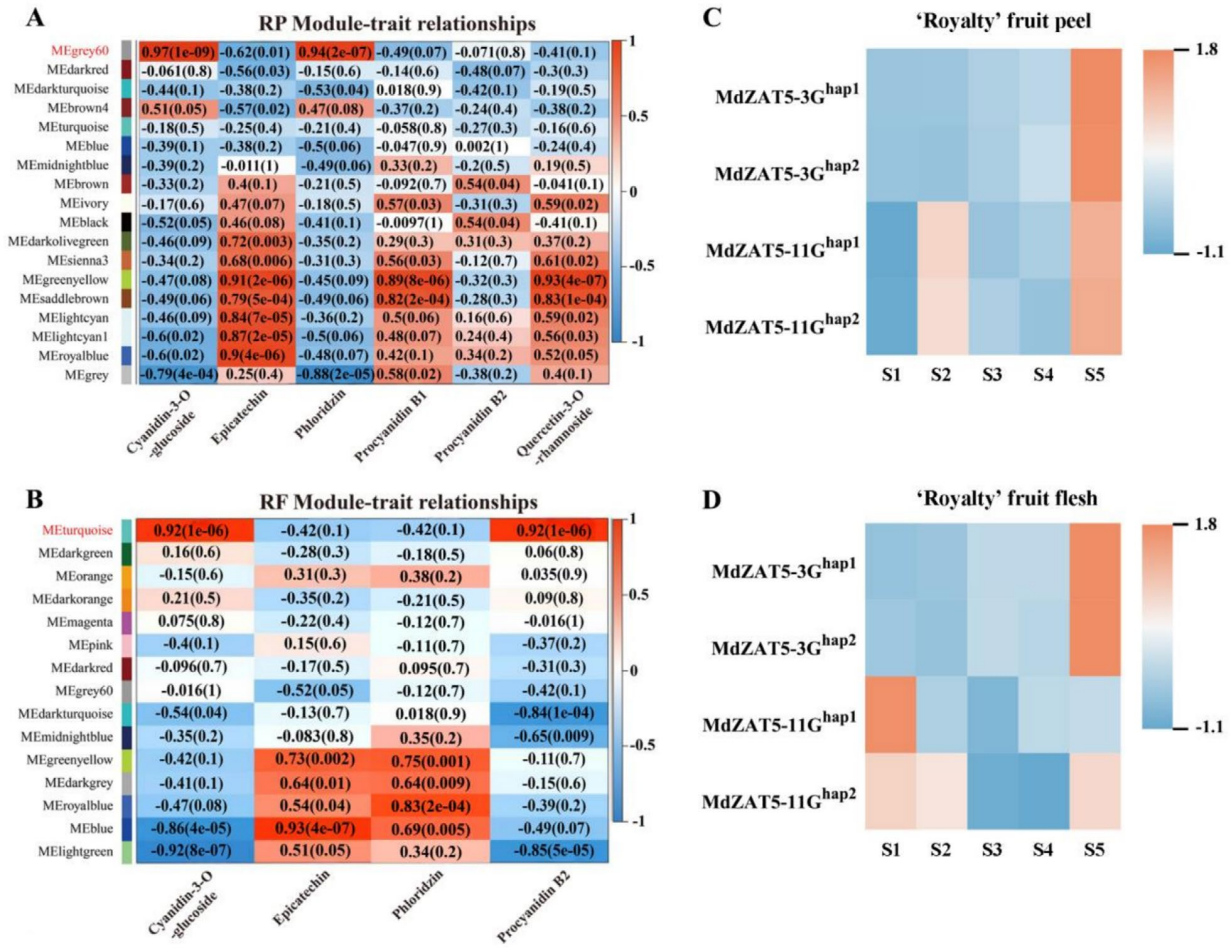
Identification of transcription factors (TFs) associated with anthocyanin biosynthesis in fruit peel and flesh using weighted gene co‐expression network analysis (WGCNA). (A) Module‐anthocyanin weight correlations and corresponding *p*‐values (in parentheses) in apple peel. The left panel shows the 18 modules. The colour scale on the right shows module‐trait correlation from −1 (green) to 1 (red). (B) Module‐anthocyanin weight correlations and corresponding *P*‐values (in parentheses) in apple flesh. The left panel shows the 15 modules. The colour scale on the right shows module‐trait correlation from −1 (green) to 1 (red). (C) Expression patterns of two MdZAT5 gene copies on each of chromosome 3 (MdZAT5‐3G) and chromosome 11 (MdZAT5‐11G), in the fruit peel at five developmental stages (S1: 45 days after flowering; S2: 75 days after flowering; S3: 105 days after flowering; S4: 135 days after flowering; S5: 165 days after flowering). (D) Expression patterns of two MdZAT5 gene copies on each of chromosome 3 (MdZAT5‐3G) and chromosome 11 (MdZAT5‐11G), in the fruit flesh at five developmental stages (S1: 45 days after flowering; S2: 75 days after flowering; S3: 105 days after flowering; S4: 135 days after flowering; S5: 165 days after flowering).

We then performed a weighted gene co‐expression network analysis (WGCNA) with anthocyanin content, resulting in 18 distinct gene modules in fruit peels (Figure 3A) and 15 modules in fruit flesh (Figure 3B), as shown in the dendrogram, where major tree branches define module groupings. Analysis of the correlation between modules and anthocyanin content revealed that the ‘MEgrey60’ (*r* = 0.86, *p* = 1e‐09) and the ‘MEturquoise’ module (*r* = 0.92, *p* = 1e‐06) were highly correlated with anthocyanin accumulation in the peel and flesh, respectively.

Co‐expression network analysis of gene expression trends in these modules further showed that 15 transcription factors in the ‘MEgrey60’ module and 4 transcription factors in the ‘MEturquoise’ module were correlated with anthocyanin biosynthesis genes (Figure S3A,B). Notably, MdZAT5 (MD03G1128800) was identified as a hub gene present in both modules. This finding suggests that MdZAT5 may be involved in the coordinated regulation of anthocyanin biosynthesis in both tissues. Genomic analysis revealed that MdZAT5 is located on chromosome 3 and chromosome 11, corresponding to haplotype 1 and haplotype 2, respectively. Transcriptome data showed that MdZAT5 exhibits significant specific expression in fruit. Four copies of MdZAT5 are highly expressed during the fruit ripening stage (Figure 3C,D).

### 2.5 Characterization of MdZAT5

Further comparative analysis revealed that the two MdZAT5 copies located on chromosome 3 (MdZAT5‐3G^hap1^ and MdZAT5‐3G^hap2^) share identical sequences (Figure S4A), while the two copies on chromosome 11 (MdZAT5‐11G^hap1^ and MdZAT5‐11G^hap2^) exhibit a sequence similarity of 98.77% (Figure S4B). In contrast, MdZAT5‐3G shows sequence similarities of 90.18% and 90.80% with MdZAT5‐11G^hap1^ and MdZAT5‐11G^hap2^, respectively (Figure S4C,D), indicating substantial divergence at the protein level.

**FIGURE 4:**
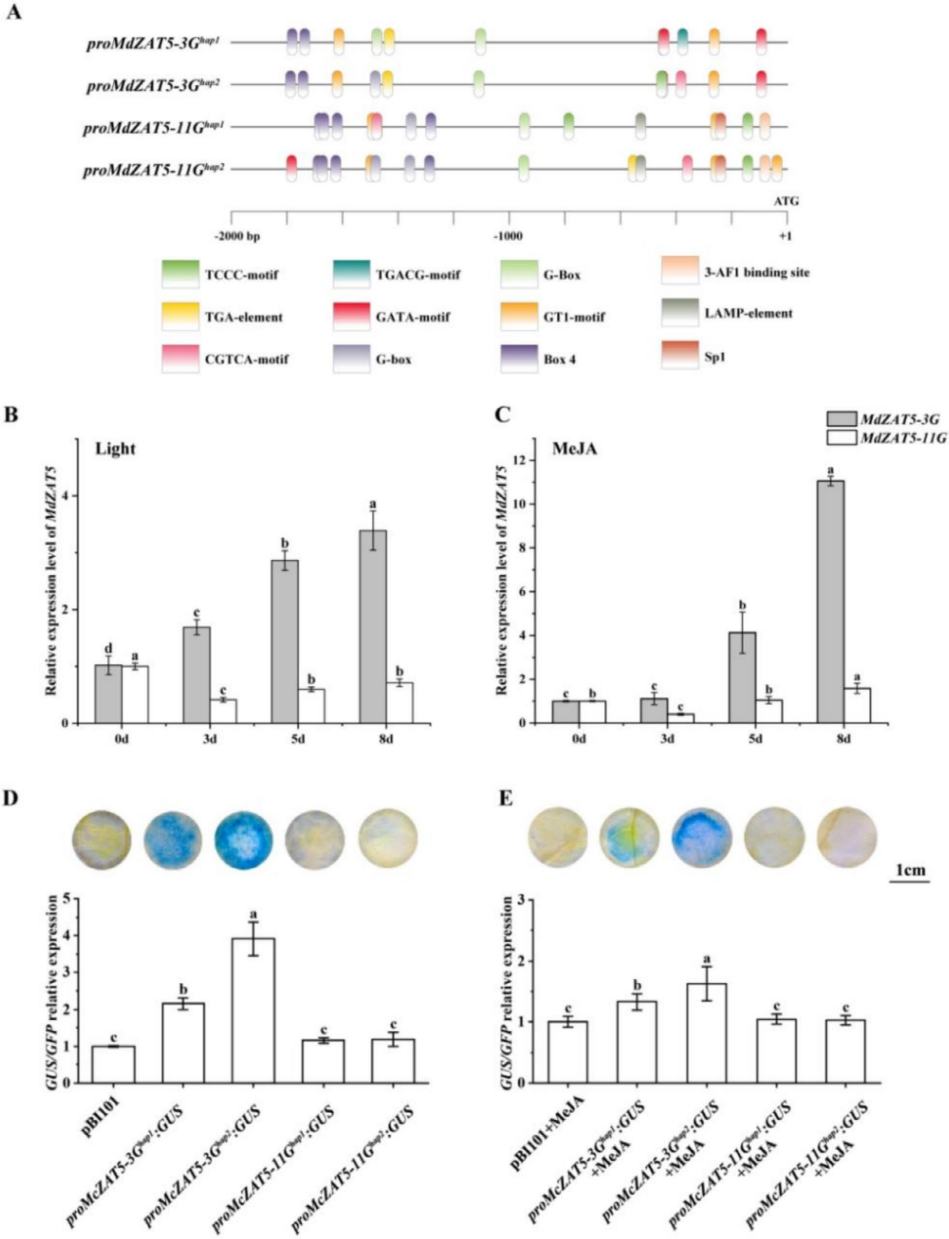
Induction characteristics of MdZAT5 in response to light and MeJA treatments. (A) Schematic overview of *cis*‐regulatory elements in the 2000‐bp promoter regions of two *MdZAT5* copies on chromosome 3 (MdZAT5‐3G) and chromosome 11 (MdZAT5‐11G). *Cis*‐elements were predicted using the PlantCARE database. (B) RT‐qPCR analysis of *MdZAT5‐3G* and *MdZAT5‐11G* transcript levels in apple calli following light treatment. (C) RT‐qPCR analysis of *MdZAT5‐3G* and *MdZAT5‐11G* transcript levels in apple calli following MeJA treatment. (D) GUS staining and quantification of *GUS/GFP* expression ratios in *Nicotiana benthamiana* leaves transiently expressing pBI101, *proMdZAT5‐3G* ^*hap1*^: *GUS*, *proMdZAT5‐3G* ^*hap2*^: *GUS*, *proMdZAT5‐11G* ^*hap1*^: *GUS*, and *proMdZAT5‐11G* ^*hap2*^: *GUS* following light treatment. (E) GUS staining and quantification of *GUS/GFP* expression ratios in *N. benthamiana* leaves transiently expressing the same constructs as in (D), following MeJA treatment. All experiments were independently repeated three times. Different letters above the bars indicate significantly different values (*p* < 0.05) calculated using one‐way analysis of variance (ANOVA) followed by Tukey's multiple range test.

To investigate the regulatory characteristics of *MdZAT5* expression, we analyzed the 2000 bp upstream promoter regions of the four *MdZAT5* copies for *cis*‐acting elements. The analysis revealed the presence of multiple light‐responsive elements (such as G‐boxes, GT1 motifs, and GATA motifs), as well as hormone‐responsive elements including auxin‐responsive elements (TGA‐element) and MeJA‐responsive elements (TGACG‐motif and CGTCA‐motif) (Figure 4A). These results suggest that *MdZAT5* transcription may be responsive to MeJA, IAA, and light.

To investigate whether the expression levels of the two gene copies are influenced by light and hormones, apple calli were subjected to light exposure and exogenous hormone treatments. RT‐qPCR analysis revealed that the expression of *MdZAT5‐3G* was progressively upregulated with increasing durations of both light and MeJA treatments (Figure 4B,C). In contrast, *MdZAT5‐11G* expression remained largely unresponsive to induction by either light or MeJA. Furthermore, following treatments with IAA, *MdZAT5‐3G* was detectable but did not exhibit consistent expression trends, whereas *MdZAT5‐11G* expression remained low or undetectable across all conditions (Figure S5).

To further determine whether these regulatory effects are mediated at the transcriptional level, we cloned the promoter sequences of *MdZAT5‐3G* ^*hap1*^, *MdZAT5‐3G* ^*hap2*^, *MdZAT5‐11G* ^*hap1*^ and *MdZAT5‐11G* ^*hap2*^ into the pBI101‐GUS‐GFP vector. GFP, driven by the *CaMV35S* promoter, was used as an internal reference to normalize infection efficiency in transient expression assays conducted in tobacco leaves. RT‐qPCR was used to quantify *GUS* and *GFP* expression, and the GUS/GFP ratio was calculated. The results showed that the promoter activities of *MdZAT5‐3G* ^*hap1*^ and *MdZAT5‐3G* ^*hap2*^ were significantly increased after light and MeJA treatments, while those of *MdZAT5‐11G* ^*hap1*^ and *MdZAT5‐11G* ^*hap2*^ remained largely unchanged (Figure 4D,E). These findings indicate that light and MeJA induce the expression of *MdZAT5‐3G*, but do not affect *MdZAT5‐11G* expression.

### 2.6 MdZAT5 Promotes Fruit Coloration Through Transcriptional Activation of Anthocyanin Biosynthetic Genes

**FIGURE 5:**
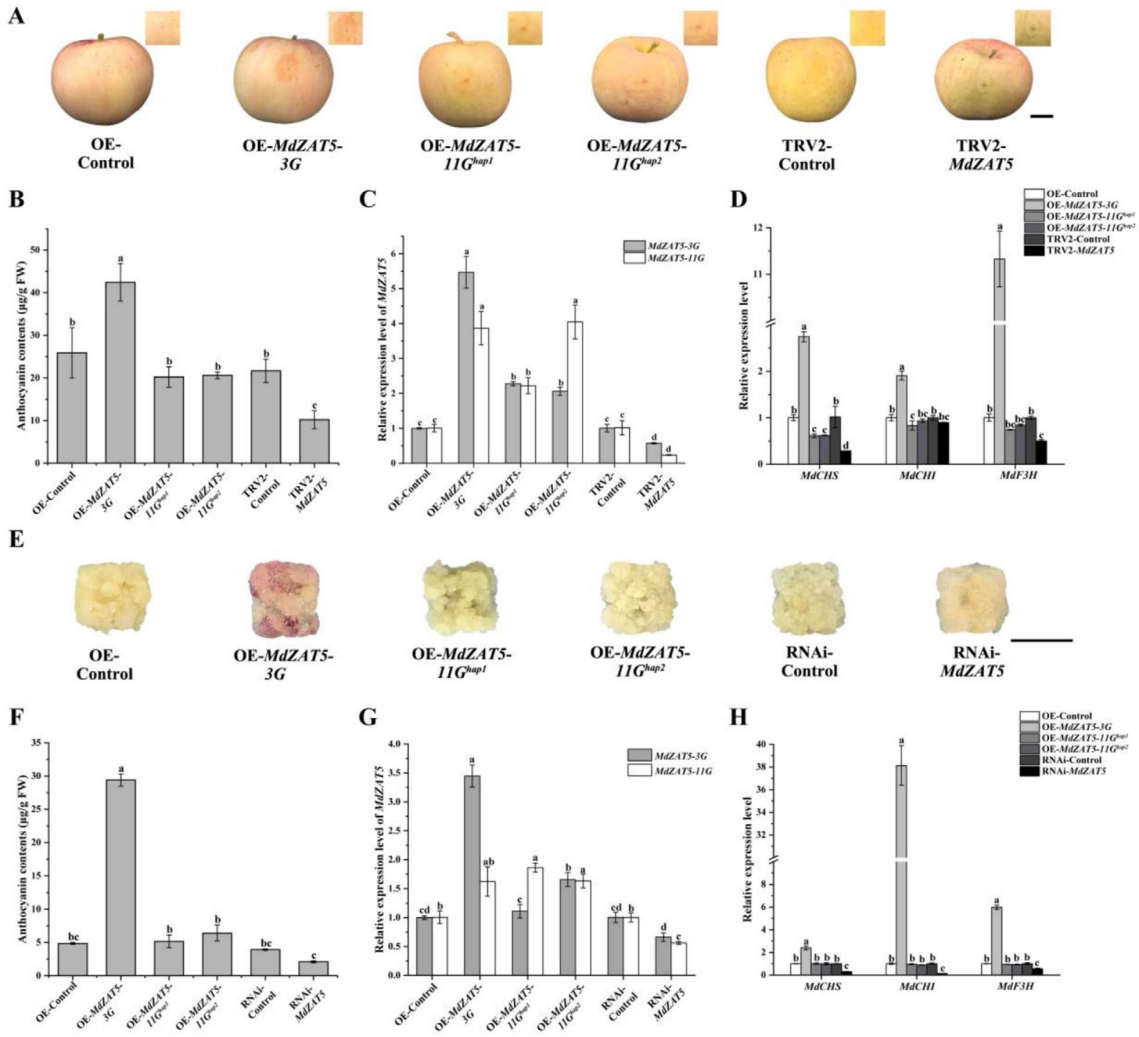
Transient transformation of MdZAT5s in apple fruit and stable transformation of MdZAT5s in apple calli. (A) Anthocyanin accumulation in apple fruit. Bagged ‘Red Fuji’ apple fruit were harvested 170 days after blooming infiltrated with pGFP‐*MdZAT5*s constructs at 5 days post‐infiltration. No anthocyanin accumulation was observed in control or *MdZAT5*‐silenced fruit. (B) Quantification of anthocyanin content in infiltrated apple fruit shown in (A). (C) Relative expression levels of MdZAT5‐3G and MdZAT5‐11G in infiltrated apple fruit, measured by RT‐qPCR. (D) Relative expression levels of anthocyanin biosynthesis genes *MdCHS*, *MdCHI* and *MdF3H* in infiltrated apple fruit, measured by RT‐qPCR. (E) Formation of pigmented transgenic cells in apple calli transformed with *MdZAT5*s under constant high light (16°C, 12 days), but not in control or *MdZAT5*‐silenced calli. (F) Anthocyanin content in transgenic apple calli shown in (D). (G) Relative expression levels of *MdZAT5‐3G* and *MdZAT5‐11G* in infiltrated apple calli, measured by RT‐qPCR. (H) Relative expression levels of anthocyanin biosynthesis genes *MdCHS*, *MdCHI* and *MdF3H* in transgenic calli determined by RT‐qPCR. Expression levels were normalized to the empty vector or wild type (set to 1). All RT‐qPCR and anthocyanin assays were performed with three biological replicates. Error bars represent the mean ± standard error (SE) of three replicates. Different letters above bars indicate statistically significant differences (*p* < 0.05), determined by one‐way ANOVA followed by Tukey's multiple range test.

To determine whether variation in *MdZAT5* expression impacts anthocyanin biosynthesis, a transient transformation was performed in apple fruit to alter its expression. The constructs pGFPGUSPLUS‐*MdZAT5‐3G* (OE‐*MdZAT5‐3G*), pGFPGUSPLUS‐*MdZAT5‐11G* ^*hap1*^ (OE‐*MdZAT5‐11G* ^*hap1*^), pGFPGUSPLUS‐*MdZAT5‐11G* ^*hap2*^ (OE‐*MdZAT5‐11G* ^*hap2*^) and suppression expression vector pTRV2‐*MdZAT5* (TRV2‐*MdZAT5*) were generated. The empty vectors pGFP and pTRV (TRV1+TRV2) were used as controls. Overexpression of *MdZAT5‐3G* significantly promoted anthocyanin accumulation compared to that in control fruit (Figure 5A). However, no obvious anthocyanin accumulation was observed in the peels of OE‐*MdZAT5‐11G* ^*hap1*^ and OE‐*MdZAT5‐11G* ^*hap2*^ fruit when compared with OE‐Control fruit (Figure 5A). Meanwhile, anthocyanin accumulation was decreased in the peels of TRV2‐*MdZAT5* infected fruit compared with those transformed with TRV2‐Control (Figure 5B). These phenotypic changes were consistent with the measured anthocyanin content in infected peel regions. To investigate the molecular basis of these changes, RT‐qPCR was conducted to analyze the expression of *MdZAT5* and anthocyanin biosynthesis genes (*MdMYB1*, *MdCHS*, *MdCHI*, *MdF3H*, *MdDFR*, *MdANS*, *MdUFGT*) in the transformed fruit (Figures 5C,D and S6). The results showed that the expression levels of *MdCHS*, *MdCHI*, and *MdF3H* followed a similar trend to that of *MdZAT5‐3G*, and in TRV2‐*MdZAT5* transformed fruit, these genes showed a significantly decreased expression pattern (Figure 5C,D). In contrast, the expression levels of anthocyanin biosynthesis genes in OE‐*MdZAT5‐11G* ^*hap1*^ and OE‐*MdZAT5‐11G* ^*hap2*^ infected fruit did not change significantly (Figure 5C,D).

To further verify the function of *MdZAT5* in fruit coloration, *MdZAT5* overexpression constructs (pGFPGUSPLUS‐*MdZAT5‐3G*, OE‐*MdZAT5‐3G*; pGFPGUSPLUS‐*MdZAT5‐11G* ^*hap1*^, OE‐*MdZAT5‐11G* ^*hap1*^; pGFPGUSPLUS‐*MdZAT5‐11G* ^*hap2*^, OE‐*MdZAT5‐11G* ^*hap2*^) and antisense suppression plasmids (pRI101RNAi‐*MdZAT5*, RNAi‐*MdZAT5*) were generated and transformed into wild‐type apple calli via *Agrobacterium*‐mediated genetic transformation. Compared with OE‐Control calli, the transformed OE‐*MdZAT5‐3G* calli exhibited visible anthocyanin accumulation, while the OE‐*MdZAT5‐11G* ^*hap1*^ and OE‐*MdZAT5‐11G* ^*hap2*^ calli retained a yellow colour (Figure 5E). At the same time, there was no significant anthocyanin accumulation in calli transformed with RNAi‐*MdZAT5*, compared with those transformed with RNAi‐Control (Figure 5E). These phenotypic observations were consistent with the measured anthocyanin content in the respective calli (Figure 5F). Expression analysis demonstrated that *MdZAT5‐3G* transcript levels were upregulated in the *MdZAT5‐3G*‐overexpression (pRI101‐*MdZAT5‐3G*) transgenic calli, while they were downregulated in the RNAi‐*MdZAT5* (pRI101RNAi‐*MdZAT5*) lines, compared with the controls (Figure 5G). RT‐qPCR analysis revealed that overexpression of *MdZAT5‐3G* enhanced anthocyanin biosynthesis by upregulating the expression of key biosynthetic genes including *MdCHS*, *MdCHI*, and *MdF3H*. Conversely, these genes showed reduced expression in the RNAi‐*MdZAT5* transgenic lines (Figures 5H and S7). Furthermore, the expression levels of *MdCHS*, *MdCHI*, and *MdF3H* were not induced by the overexpression of *MdZAT5‐11G* ^*hap1*^ and *MdZAT5‐11G* ^*hap2*^ (Figure 5H). Together, these data suggest that *MdZAT5‐3G* functions as a positive regulator of anthocyanin accumulation by activating anthocyanin biosynthesis genes, whereas *MdZAT5‐11G* ^*hap1*^ and *MdZAT5‐11G* ^*hap2*^ may lack this regulatory capability and are potentially not involved in fruit coloration. Accordingly, we concentrated our further investigations on *MdZAT5‐3G* due to its role in anthocyanin regulation.

To verify that MeJA promotes the expression of *MdZAT5‐3G* in the flesh, we treated OE‐*MdZAT5‐3G* calli with MeJA. The results showed that MeJA significantly enhanced anthocyanin accumulation in the transgenic calli by upregulating *MdZAT5‐3G* expression. Moreover, the expression of key anthocyanin biosynthetic genes (*MdCHS*, *MdCHI*, and *MdF3H*) was also markedly increased in response to the elevated expression of *MdZAT5‐3G* (Figure S8).

### 2.7 MdZAT5 Directly Binds to the Promoters of Anthocyanin Biosynthesis Genes

**FIGURE 6:**
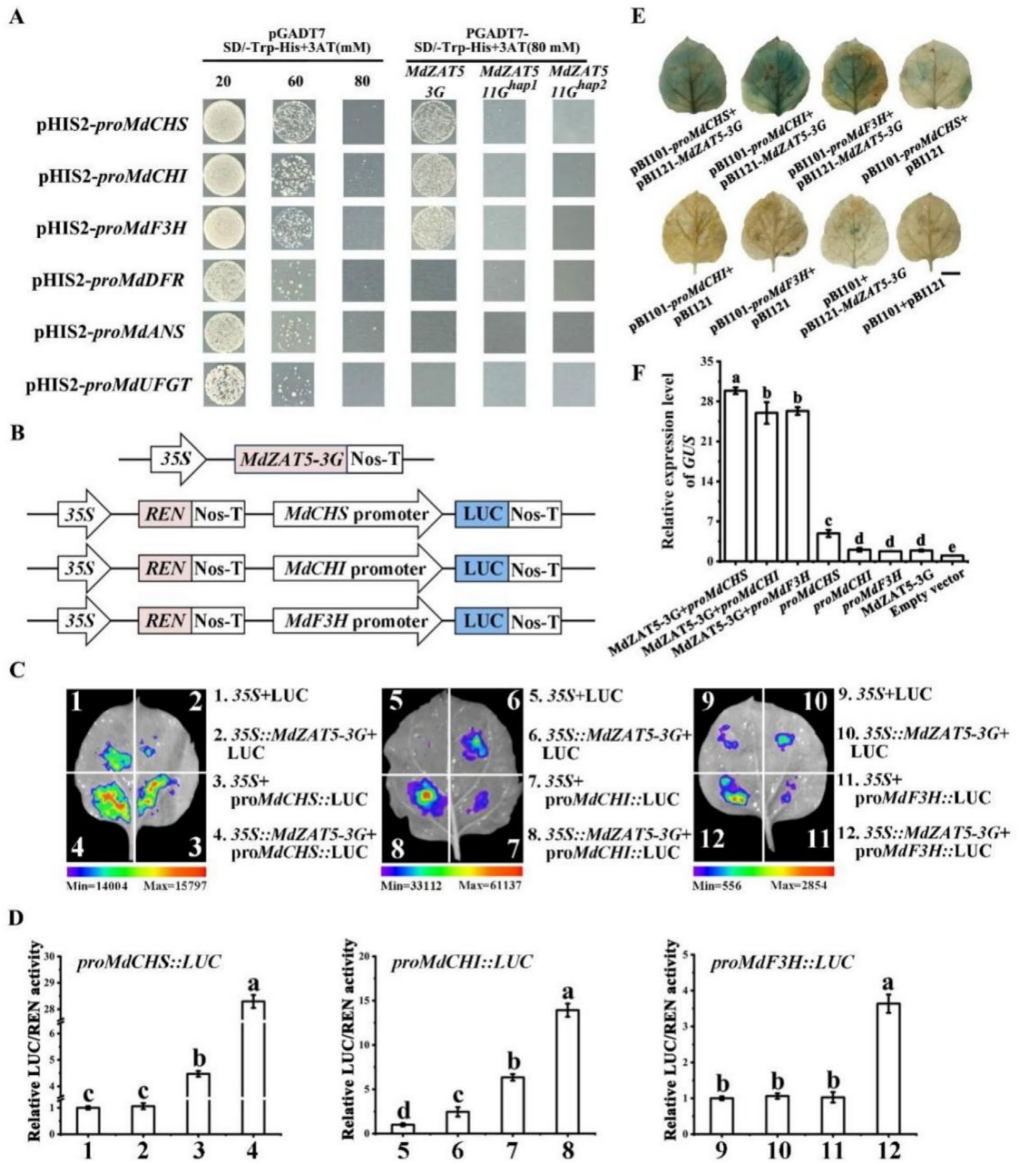
MdZAT5 proteins directly bind to the promoters of anthocyanin biosynthesis genes and activate their transcription. (A) Yeast one‐hybrid assays indicated that MdZAT5 proteins directly bind to the promoters of *MdCHS*, *MdCHI*, and *MdF3H*. Yeast cells co‐transformed with pGADT7 + pHIS2‐*ProMdCHS*, pGADT7 + pHIS2‐*ProMdCHI* or pGADT7 + pHIS2‐*ProMdF3H* were used as negative controls. Numbers in parentheses indicate the optimal 3‐amino‐1,2,4‐triazole (3‐AT) concentrations used for suppressing background HIS3 expression. (B) Schematic representation of the dual‐luciferase reporter system: LUC reporter constructs containing the promoters of *MdCHS*, *MdCHI*, and *MdF3H*, and an effector construct expressing MdZAT5‐3G under the 35S promoter. (C) Representative images of *Nicotiana benthamiana* leaves 72 h after transient co‐infiltration, showing MdZAT5‐3G‐induced activation of anthocyanin‐related promoters. (D) Quantification of LUC activity in *N. benthamiana* leaves co‐expressing MdZAT5‐3G and LUC reporter constructs driven by the *MdCHS*, *MdCHI*, or *MdF3H* promoters. (E) GUS reporter assay in tobacco leaves co‐expressing MdZAT5‐3G and the promoters of *MdCHS*, *MdCHI*, and *MdF3H*. GUS staining was observed only in the co‐infiltrated samples. Scale bar = 1 cm. (F) Quantification of *GUS* expression by RT‐qPCR. Error bars represent the mean ± standard error (SE) of six biological replicates. Different letters above the bars indicate statistically significant differences (*p* < 0.05), as determined by one‐way ANOVA followed by Duncan's multiple range test.

To explore the mechanism of *MdZAT5*‐mediated anthocyanin accumulation in apple fruit, yeast one hybrid (Y1H) assays were conducted to verify the binding of *MdZAT5* to the promoters of anthocyanin biosynthesis genes. The full‐length of *MdZAT5‐3G*, *MdZAT5‐11G* ^*hap1*^ and *MdZAT5‐11G* ^*hap2*^ gene was inserted into the pGADT7 vector, and the promoters of anthocyanin biosynthesis genes *MdCHS*, *MdCHI*, *MdF3H*, *MdDFR*, *MdANS*, and *MdUFGT* were incorporated into the pHIS2 vector. The Y1H results indicated that MdZAT5‐3G directly binds to the promoters of *MdCHS*, *MdCHI*, and *MdF3H* (Figure 6A). To determine whether MdZAT5‐3G can activate the transcription of these genes, the promoters of *MdCHS*, *MdCHI*, and *MdF3H* were fused with the LUC reporter gene (Figure 6B). The results showed that co‐transformation of *35S*: *MdZAT5‐3G* and *proMdCHS*: LUC, *proMdCHI*: LUC and *proMdF3H*: LUC significantly increased luminescence intensity, respectively (Figure 6C,D). We next investigated the transcriptional regulation of *MdCHS*, *MdCHI*, and *MdF3H* by MdZAT5‐3G using a transient transactivation assay in tobacco leaves. A construct containing the MdZAT5^3G^ coding sequence driven by the cauliflower mosaic virus *35S promoter* was co‐transformed into tobacco leaves along with constructs harbouring the promoters of *MdCHS*, *MdCHI*, and *MdF3H* fused to a *GUS* reporter gene. Co‐expressing *MdZAT5‐3G* with the *MdCHS*, *MdCHI*, and *MdF3H* promoters enhanced GUS staining and significantly increased the relative transcriptional activity of *GUS* (Figure 6E,F).

### 2.8 Interaction Between MdZAT5 and MdHY5 Proteins

**FIGURE 7:**
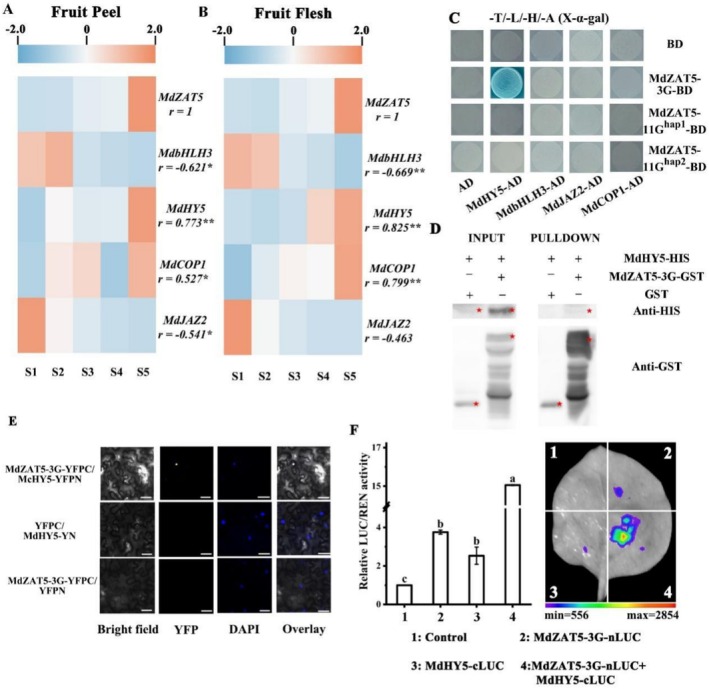
Interaction between MdZAT5 and MdHY5 proteins. (A) FPKM values of potential MdZAT5‐interacting proteins in apple fruit peel in five developmental stage (S1: 45 days after flowering; S2: 75 days after flowering; S3: 105 days after flowering; S4: 135 days after flowering; S5: 165 days after flowering). (B) FPKM values of potential MdZAT5‐interacting proteins in apple fruit flesh in five developmental stage (S1: 45 days after flowering; S2: 75 days after flowering; S3: 105 days after flowering; S4: 135 days after flowering; S5: 165 days after flowering). (C) Yeast two‐hybrid assay showing that four candidate proteins interact with MdZAT5, as indicated by blue colonies. The empty pGADT7 vector was used as a negative control. (D) In vitro pull‐down assay showing the interaction between MdZAT5‐3G and MdHY5. GST‐tagged MdZAT5‐3G was immobilized on glutathione sepharose beads and incubated with HIS‐tagged MdHY5. Interacting proteins were detected by immunoblotting using anti‐GST and anti‐HIS antibodies. (E) Bimolecular fluorescence complementation (BiFC) assay in *Nicotiana benthamiana* leaves showing the interaction between MdZAT5‐3G and MdHY5. Yellow fluorescence indicates a positive interaction. Scale bar = 25 μm. (F) Dual‐luciferase assay detecting the interaction between MdZAT5‐3G and MdHY5 based on LUC/REN activity in *N. benthamiana* leaves. Different letters above the bars indicate statistically significant differences (*p* < 0.05), determined by one‐way ANOVA followed by Duncan's multiple range test. Error bars represent the mean ± standard error (SE) of replicate measurements.

To further understand the molecular network of MdZAT5, we conducted transcriptome analysis to examine the expression levels of anthocyanin related and MeJA response TFs during fruit development in apple peels and flesh. The results showed that *MdHY5*, *MdCOP1*, and *MdMYC2* have similar expression variation during apple fruit development with *MdZAT5‐3G*, we therefore investigated the interaction between MdZAT5‐3G and these TFs (Figure 7A,B). Y2H assays were conducted to examine whether MdZAT5‐3G directly interacted with these TFs. Our result showed that MdZAT5‐3G interacted with MdHY5 in yeast. And to verify the interaction between MdZAT5‐3G and MdHY5, an in vitro pull‐down analysis was performed. The recombinant MdZAT5‐3G‐GST protein and the GST control were incubated in vitro with the recombinant MdHY5‐6×HIS protein. The protein was eluted with glutathione and immunoblotted with anti‐GST and anti‐HIS antibody. The results showed that MdHY5‐6×HIS was pulled down by MdZAT5‐3G‐GST, but GST alone was not, indicating that MdZAT5‐3G interacted directly with the MdHY5 in vitro (Figure 7D).

Furthermore, we carried out bimolecular fluorescence complementation (BiFC) assays. Full length MdZAT5‐3G and MdHY5 were cloned into pSPYNE‐35S/pUC‐SPYNE or pSPYCE‐35S/pUC‐SPYCE to generate MdZAT5‐3G‐YFPN, and MdHY5‐YFPC. Fluorescence microscopy observation demonstrated that MdZAT5 interacted with MdHY5 in the nucleus (Figure 7E). In addition, an in vivo luciferase complementation imaging assays were also conducted. MdZAT5‐3G was cloned into a pCAMBIA1300‐nLUC vector, and MdHY5 was fused to a pCAMBIA1300‐cLUC vector. Consistent with the results of above binding assays, the fluorescence signal was observed when MdZAT5‐3G was coexpressed with MdHY5 (Figure 7F). We also detected the expression of *MdHY5* under light and MeJA treatments in apple peel and flesh. In apple fruit peels, *MdHY5* expression was induced by light but not by MeJA; in contrast, in apple calli, *MdHY5* expression was upregulated in response to MeJA treatment (Figure S9). These results suggest that MdZAT5‐3G may functionally interact with MdHY5 to coordinate the regulation of anthocyanin accumulation in response to light and MeJA signals in fruit peel and flesh tissues.

### 2.9 MdHY5 Promotes the Binding of MdZAT5 to Its Target Genes

**FIGURE 8:**
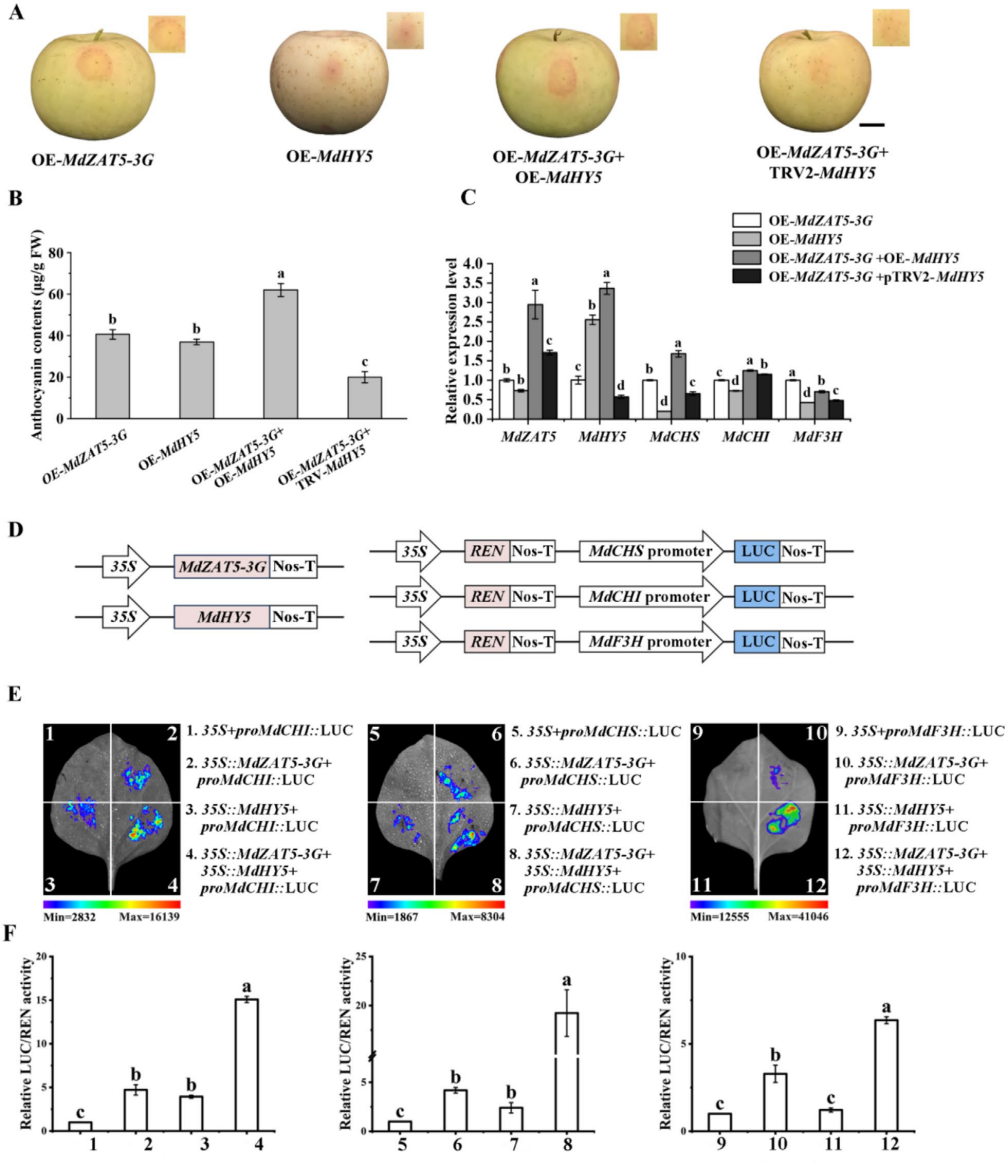
Co‐expression of *MdZAT5‐3G* and *MdHY5* promotes anthocyanin accumulation in apple and activates the expression of downstream promoters. (A) Coloration of apple fruit peel around injection sites after transient transformation. Full‐length cDNAs of MdZAT5‐3G and MdHY5 were cloned into the pGFP vector for overexpression, while gene fragments were inserted into the TRV vector for silencing. Scale bar = 1 cm. (B) Anthocyanin content in apple fruit samples shown in (A). (C) Relative expression levels of *MdZAT5‐3G*, *MdHY5*, and anthocyanin‐related genes in transformed apple fruit, measured by RT‐qPCR. Expression levels were normalized to the expression of OE‐*MdZAT5‐3G* (set to 1). (D) Schematic representation of dual‐luciferase reporter and effector vectors: The promoters of *MdCHS*, *MdCHI*, and *MdF3H* were fused to the LUC reporter, while MdZAT5‐3G and MdHY5 were cloned into the effector vector under control of the 35S promoter. (E) Representative images of *Nicotiana benthamiana* leaves 72 h after co‐infiltration, showing that co‐expression of *MdZAT5‐3G* and *MdHY5* activates anthocyanin‐related gene promoters. (F) Relative LUC activity from transient expression assays of the *MdCHS*, *MdCHI*, and *MdF3H* promoters co‐infiltrated with MdHY5 and/or MdZAT5‐3G effectors LUC assays were performed with three independent biological replicates. All RT‐qPCR, anthocyanin assays and relative LUC activity were performed with three biological replicates. Error bars represent the mean ± standard error (SE) of three replicates. Different letters above bars indicate statistically significant differences (*p* < 0.05), determined by one‐way ANOVA followed by Tukey's multiple range test.

Since the MdHY5 interacts with MdZAT5‐3G protein and both function as regulators of anthocyanin accumulation, we examined the effects of the interaction between these two proteins on the transcription of its target genes, and *MdHY5* was overexpressed or silenced in the OE‐*MdZAT5‐3G* infiltrated fruit. Anthocyanin accumulation was observed in the fruit expressing both the OE‐*MdZAT5‐3G* and OE‐*MdHY5* constructs, and significantly higher than the fruit expressing only the OE‐*MdZAT5‐3G* or OE‐*MdHY5* construct (Figure 8A,B). Silencing of *MdHY5* significantly suppressed anthocyanin accumulation in OE‐*MdZAT5‐3G* fruit. RT‐qPCR analysis also verified the expressions of anthocyanin biosynthetic genes *MdCHS*, *MdCHI*, and *MdF3H* showed much higher expressions in the OE‐*MdZAT5‐3G* and OE‐*MdHY5* co‐infiltrated fruit, and silencing *MdHY5* led to lower expression of biosynthesis genes involved in anthocyanin accumulation in OE‐*MdZAT5‐3G* fruit (Figure 8C).

Furthermore, a dual‐luciferase assay was performed to determine the effect of the *MdZAT5‐3G*‐*MdHY5* complex on target gene transcription. *MdZAT5‐3G* and *MdHY5* were expressed separately under the control of *35S* promoters on the pGreenII 62‐SK plasmid vector as effectors, and the luciferase gene was used as a reporter behind the *MdCHS*, *MdCHI* or *MdF3H* promoters in the pGreenII 0800‐LUC plasmid vector (Figure 8D). The MdZAT5‐3G‐MdHY5 complex significantly affected the ability of *MdZAT5‐3G* to bind to its target gene promoters. *MdZAT5‐3G* alone was able to drive the expression of the luciferase gene, and when the constructs expressing both proteins were present, luciferase expressions were elevated significantly (Figure 8E,F).

### 2.10 Structural Differences Between Different Copies of MdZAT5

**FIGURE 9:**
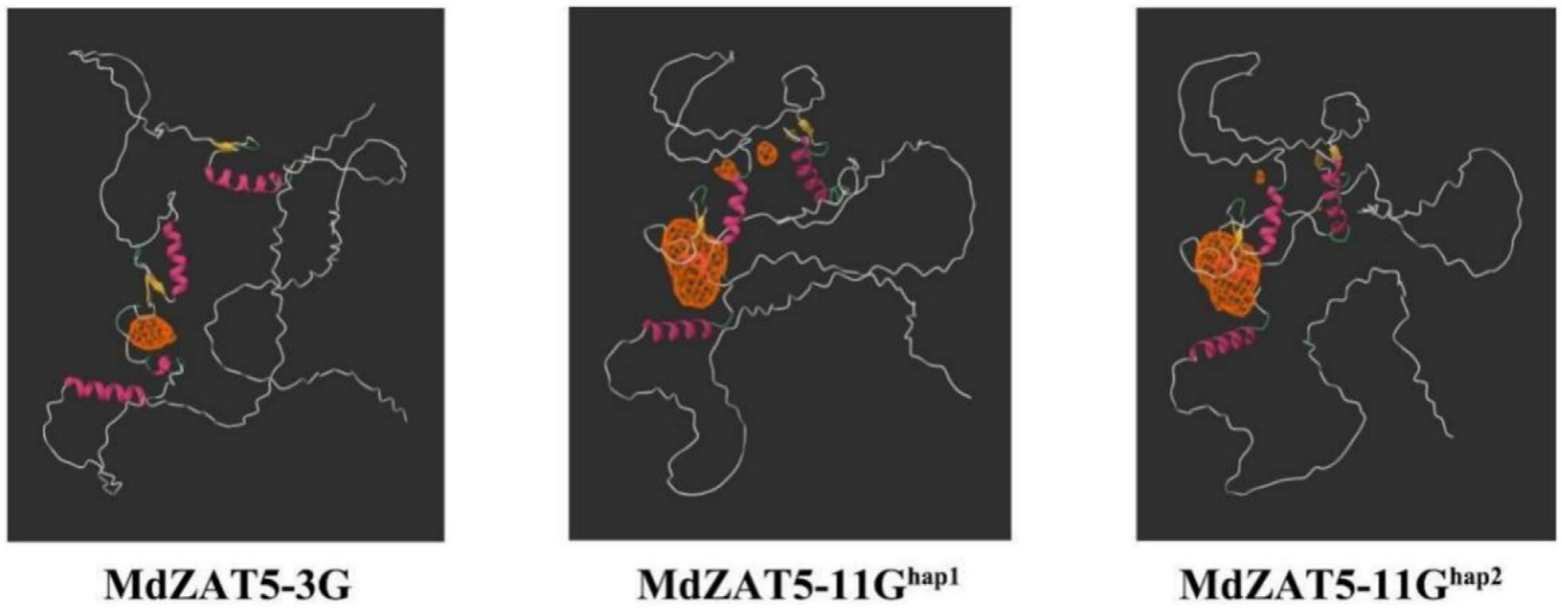
Predicted three‐dimensional structures and binding pockets of MdZAT5‐3G, MdZAT5‐11G^hap1^ and MdZAT5‐11G^hap2^. Protein structures were predicted using AlphaFold3, and potential ligand‐binding pockets were identified using DeepSite. Orange regions indicate the predicted binding pocket locations.

Above studies revealed that *MdZAT5‐3G* can interact with MdHY5 and synergistically enhance its ability to activate the promoters of *MdCHS*, *MdCHI*, and *MdF3H*, thereby promoting anthocyanin accumulation in fruit. However, this regulatory function is absent in MdZAT5‐11G^hap1^ and MdZAT5‐11G^hap2^. To explore the possible reasons for this difference, we predicted the three‐dimensional structures of MdZAT5‐3G, MdZAT5‐11G^hap1^ and MdZAT5‐11G^hap2^ proteins using AlphaFold (https://alphafoldserver.com/) (Abramson et al. 2024) (Figure 9). The results showed that the protein structures of MdZAT5‐11G^hap1^ and MdZAT5‐11G^hap2^ were highly similar, while MdZAT5‐3G exhibited notable structural differences compared to the other two proteins (Figure 9).

Since protein structure affects its ability to bind DNA or other proteins, we further utilized the AlphaFold predictions to perform protein‐binding pocket prediction using DeepSite (https://open.playmolecule.org/tools/deepsite), selecting results with a confidence score above 0.8 (Jiménez et al. 2017) (Figures 9 and S10). The analysis revealed that MdZAT5‐11G^hap1^ and MdZAT5‐11G^hap2^ shared similar binding pocket distributions, whereas MdZAT5‐3G displayed a distinct and unique binding pocket profile. These results suggest that the differences in binding pocket architecture may lead to altered molecular interaction properties in MdZAT5‐3G, thereby contributing to its specific regulatory function.

## 3 Discussion

Anthocyanin accumulation is a key determinant of the appearance quality of apple fruit. The anthocyanin biosynthesis pathway has been well characterized in various plants, including grape, pear, and apple. It is well established that the MYB‐bHLH‐WD repeat (MBW) protein complex plays a central role in regulating anthocyanin biosynthesis (Ramsay and Glover 2005; Allan et al. 2008). Among these components, MYB transcription factors (TFs) are recognized as crucial regulators of anthocyanin accumulation (Allan et al. 2008). In apple, MdMYB1, MdMYB10, and MdMYBA have been identified as positive regulators of anthocyanin biosynthesis and contribute to the coloration of both fruit peels and flesh (Takos et al. 2006; Ban et al. 2007; Espley et al. 2007). bHLH proteins, as important components of the MYB‐bHLH‐WD40 complex, also play a significant role in regulating anthocyanin biosynthesis. Specifically, MdbHLH3 and MdbHLH33 interact with MYB proteins to modulate anthocyanin accumulation (Espley et al. 2007). While many studies have focused on anthocyanin accumulation in apple fruit peels or flesh, the coordinated regulatory mechanisms underlying fruit coloration development still require further investigation.

Biological function analysis reveals that zinc‐finger proteins are widely involved in plant growth and development, flowering time and stress resistance. Recently, several researches showed that cysteine2/histidine2‐type transcription factor ZINC FINGER of *Arabidopsis thaliana* can be candidate anthocyanin regulator. In *Arabidopsis*, *ZAT6* was significantly activated after exogenous H_2_O_2_ treatment, and *AtZAT6* directly activated the expressions of anthocuanin related TFs and positively affected the concentrations of both anthocyanin and total flavonoids (Shi et al. 2018). In peach, PpBBX32 and PpZAT5 are upstream activators of PpMYB10.1, allowing JAs to participate in temperature‐dependent and tissue‐specific anthocyanin accumulation (Huang et al. 2024). In pear, PpZAT5 could directly bind to CAAT motif of PpBBX18 promoter and inhibit the anthocyanin biosynthesis by repressing PpBBX18 expression (Zhang et al. 2022). These researches showed that Zinc finger protein also play important role in anthocyanin accumulation in plants. In this study, the overarching goal was to select TFs associated with the regulation of anthocyanin accumulation in an ever‐red fruit crabapple cultivar ‘Royalty’. Transcriptome analysis of different apple peels and flesh development stages identified a zinc‐finger protein, ZAT5, was correlated with fruit coloration during fruit peel and flesh coloration development. We also construct haplotype‐resolved genome sequences of this special crabapple cultivar. Genome sequencing analysis showed that MdZAT5 has two copies on chromosomes 3 and 11, respectively, and displayed significant specific expression in fruit. Overexpression of *MdZAT5‐3G* in apple calli and fruit promote anthocyanin biosynthesis and the opposite results were detected in *MdZAT5* suppressed fruit and calli. So *MdZAT5‐3G* was characterized to be the key regulatory gene for enhanced anthocyanin biosynthesis in the apple fruit. Previous studies have demonstrated that *MdMYB10*/*MYB1* are key transcription factors that directly regulate anthocyanin biosynthetic genes. At the same time, different transcription factors may function under distinct environmental or inductive conditions to fine‐tune anthocyanin accumulation. Based on our findings, we hypothesize that MdZAT5‐3G may cooperate with MdMYB10/MYB1 to regulate anthocyanin accumulation in apple fruit under different developmental or environmental contexts.

ELONGATED HYPOCOTYL 5 (HY5) is a basic domain/Leu zipper TF, and have been identified as the key components in the light signalling pathway, which located downstream of *COP1* that is degraded by *COP1* in darkness and induced under light condition (Osterlund et al. 2000; Oravecz et al. 2006). HY5 is involved in fundamental developmental processes in plants, including cell proliferation, cell elongation, and chloroplast development. Interestingly, HY5 also regulates the accumulation of anthocyanin in plants. In tomato ( *Solanum lycopersicum* ) fruit, genes involved in anthocyanin biosynthesis were revealed as direct targets of *SlHY5* by chromatin immunoprecipitation, and loss of function of tomato HY5 impairs pigment accumulation (Wang et al. 2021). Furthermore, *MdHY5* promoted anthocyanin accumulation by directly regulating expression of the *MdMYB10* gene and downstream anthocyanin biosynthesis genes (An et al. 2017). Meanwhile, HY5 also regulating anthocyanin biosynthesis by interacting with other anthocyanin regulators. *MdBBX22* interacts with *MdHY5* and enhances the binding of *MdHY5* to the promoters of *MdMYB10* and *MdCHS* through directly interacting with *MdHY5*, thus to promote anthocyanin biosynthesis under UV‐B treatment (An, Wang, et al. 2019). Low temperature induced *MdbHLH3* could directly bind to the promoter of *MdBBX20* and that *MdBBX20* could interact with *MdHY5* to synergistically regulate the expression of *MdMYB1*, *MdANS* and *MdDFR* to promote anthocyanin accumulation (Fang et al. 2019). Our results showed that *MdZAT5‐3G* could interact with *MdHY5* and significantly higher anthocyanin accumulation was observed in OE‐*MdZAT5‐3G* and OE‐*MdHY5* constructs co‐infiltrated apple fruit. Furthermore, *MdZAT5‐3G* and *MdHY5* interaction enhances the binding ability of *MdZAT5‐3G* to the *MdCHS*, *MdCHI* and *MdF3H* promoters. So we deduced that *MdZAT5‐3G* regulates fruit coloration depends on the activity of MdHY5. Meanwhile, expression analysis also showed that *MdZAT5‐3G* and *MdHY5* highly expressed in fruit peels by light treatment, suggested that *MdZAT5‐3G* and *MdHY5* response to light and regulates anthocyanin accumulation in fruit peels. Based on these results, we deduce that MdHY5 enhances MdZAT5‐3G's DNA binding activity to anthocyanin biosynthetic gene promoters. In addition, MdHY5 may further support MdZAT5‐3G function by promoting its nuclear localization or stabilizing the protein, we will conduct further research in the future.

The plant hormone jasmonate (JA) is involved in many plant developmental processes including modulation of trichome initiation, root regeneration, leaf senescence, biosynthesis of secondary metabolites and biotic and abiotic stresses (Gaquerel and Stitz 2017; Wasternack and Hause 2013). Many studies involving various species also have been performed to elucidate the role of JA in fruit ripening (Concha et al. 2013; Fan et al. 1997; Saniewski et al. 1987; Zhou et al. 2019). Recently study showed that MeJA treatment induced the expression of JA signalling pathway transcription factor MdMYC2, MdMYC2 directly bound to the promoters of both *MdACS1* and the ACC oxidase gene *MdACO1* and enhanced their transcription and promotes ethylene biosynthesis in apple fruit (Li et al. 2017). Furthermore, JA is essential in modulating the accumulation of anthocyanin in apple. JA signalling repressor *MdJAZ1* interacted with *MdTRB1* (telomere‐binding protein) and interfered with the interaction between *MdTRB1* and *MdMYB9*, therefore negatively modulating *MdTRB1*‐promoted biosynthesis of anthocyanin (An et al. 2021). And *MdMYB24L* response to JA signal and promote anthocyanin accumulation by interacting with JA signalling factors (*MdJAZ8*, *MdJAZ11*, and *MdMYC2*) and binds to the promoters of *MdUFGT* and *MdDFR* (Wang et al. 2019). Meanwhile, JA treatment also increase the expression of HY5 in *Arabidopsis* and apple (Prasad et al. 2012; Ortigosa et al. 2020). And our data suggested that several JA related *cis*‐elements were detected in the promoter of *MdZAT5*, and the expression analysis showed that the JA treatment significantly induced the expression of *MdZAT5* and *MdHY5* in calli, but not in fruit peels, which suggested that *MdZAT5* may as an anthocyanin regulator, located at the downstream of JA signalling pathway in fruit flesh.

Genome‐wide duplication (GWD) plays a significant role in plant diversification and evolution (Soltis et al. 2009). Sequencing of the apple genome revealed that a relatively recent (over 50 million years ago) GWD event caused the ancestor of the *Malus* genus to evolve from 9 chromosomes to 17. Pairwise comparisons of the 17 apple chromosomes revealed strong collinearity between chromosomes 3 and 11, 5 and 10, 9 and 17, and 13 and 16, suggesting that each pair primarily originated from a single ancestral chromosome. Small‐scale interchromosomal rearrangements have occurred among these chromosomes during evolution. These GWD events have driven gene family expansion by generating numerous duplicated genes, thereby providing a genetic basis for functional innovation through mechanisms such as gene dosage effects, neofunctionalization, and subfunctionalization (Velasco et al. 2010). In our previous study, the homologous genes MdMPK4‐06G and MdMPK4‐14G show 96% sequence identity. During the fruit colouring stage, daylight activates *MdMPK4‐06G*, which phosphorylates MdMYB1, stabilizing its protein structure and enhancing its transcriptional activity, thereby upregulating genes involved in the anthocyanin biosynthesis pathway in the fruit peel. At night, the highly expressed MdMPK4‐14G phosphorylates MdERF17, which in turn activates downstream gene expression and promotes fruit degreening. The synergistic effect of *MdMPK4‐06G* and *MdMPK4‐14G* facilitates daytime fruit colouring and nighttime chlorophyll degradation, highlighting the widespread presence of copy‐specific functional divergence in woody plants and its critical role during fruit development (Wang et al. 2022; Yang et al. 2021).

Although the anthocyanin biosynthetic pathway in apple has been extensively studied, it is well established that different transcription factors can regulate distinct subsets of pathway genes. For example, MdWRKY40 promotes MdMYB1‐mediated activation of downstream *MdDFR* and *MdUFGT* promoters (An, Zhang, et al. 2019), while MdHY5 specifically activates *MdCHI* and *MdUFGT* promoters to enhance anthocyanin accumulation (Xing et al. 2023). These examples suggest that MdZAT5‐3G may preferentially regulate a subset of upstream pathway genes (e.g., *MdCHS*, *MdCHI*, *MdF3H*), whereas other transcription factors, including MYBs and HY5, may control downstream biosynthesis genes.

In this study, we assembled a haplotype‐resolved genome of *Malus* crabapple ‘Royalty’ and identified two MdZAT5 gene copies located on chromosomes 3 and 11, respectively, which play important roles in fruit coloration. Among them, MdZAT5‐3G activates the promoters of *MdCHS*, *MdCHI*, and *MdF3H*, thereby regulating anthocyanin accumulation in the fruit through interaction with MdHY5. In contrast, MdZAT5‐11G^hap1^ and MdZAT5‐11G^hap2^ lack this regulatory function. Protein structural prediction and binding site analysis using AlphaFold and DeepSite revealed significant differences in the protein‐binding pockets of MdZAT5‐3G compared to those of MdZAT5‐11G^hap1^ and MdZAT5‐11G^hap2^. We hypothesize that these structural differences underlie the functional divergence among the gene copies. This divergence likely originated from chromosomal rearrangements during evolution, leading to condition‐specific functional loss in the MdZAT5‐11 copies.

**FIGURE 10:**
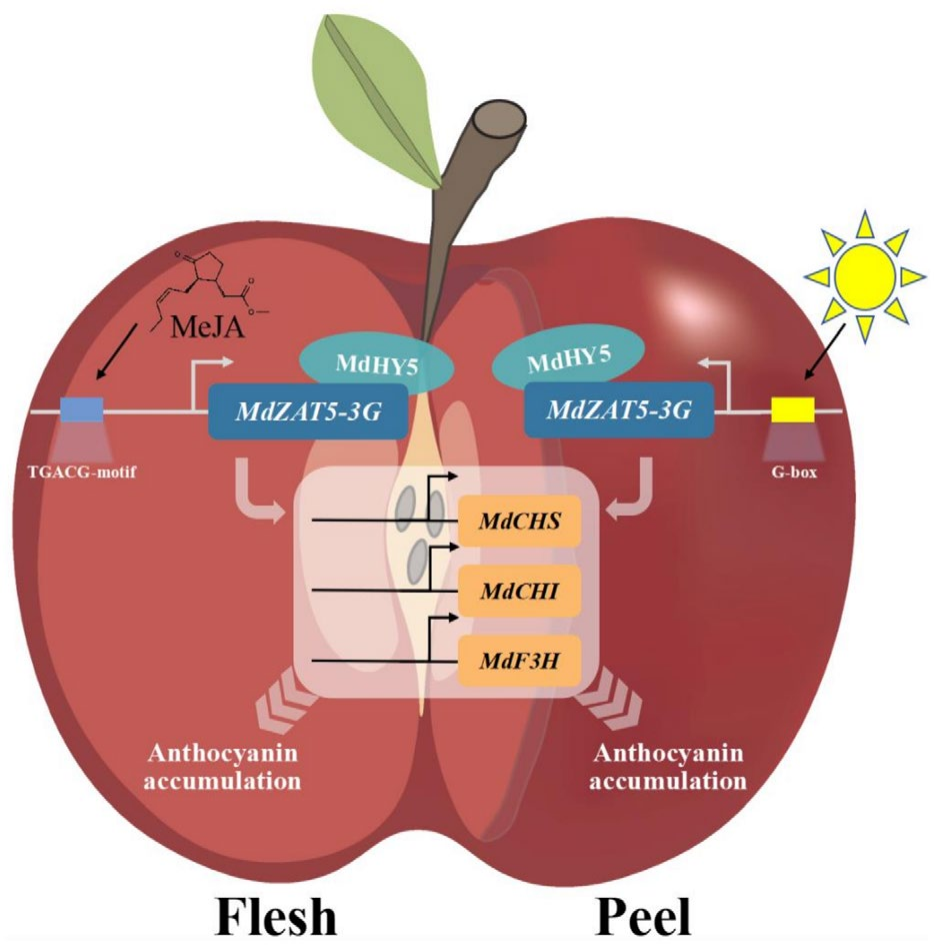
Proposed model of MdZAT5‐3G‐mediated regulation of anthocyanin biosynthesis in apple fruit peel and flesh in response to light and methyl jasmonate (MeJA) signals. During fruit ripening, MeJA induces the expression of both *MdZAT5‐3G* and *MdHY5* in the fruit flesh, while light induces their expression in the fruit peel. The MdZAT5‐3G and MdHY5 proteins interact to form a transcriptional complex that enhances anthocyanin accumulation by promoting the activation of *MdCHS*, *MdCHI*, and *MdF3H* gene promoters.

Therefore, we propose a model in which MdZAT5‐3G coordinately regulates anthocyanin biosynthesis in apple fruit peel and flesh in response to light and Methyl‐jasmonic acid (MeJA) induction (Figure 10). During fruit ripening, JA induces the expression of both *MdZAT5*‐*3G* and *MdHY5* in the fruit flesh, while light induces their expression in the fruit peel. *MdZAT5*‐*3G* and *MdHY5* then form a protein complex that positively modulates anthocyanin accumulation by enhancing the transactivation activity of *MdZAT5*‐*3G* on the promoters of *MdCHS*, *MdCHI*, and *MdF3H*.

## 4 Conclusion

A haplotype‐resolved genome sequence of high anthocyanin content model cultivar *Malus* cv. ‘Royalty’ was generated. Our study demonstrated that *MdZAT5‐3G* and *MdHY5* are activated by light in fruit peel and Methyl‐jasmonic acid (MeJA) in fruit flesh, and form a protein complex that positively modulates anthocyanin accumulation. This complex enhances the transactivation ability of *MdZAT5‐3G* on the promoters of anthocyanin biosynthesis genes in apple fruit (Figure 10). These findings broaden and deepen our understanding of the molecular mechanisms underlying fruit peel and flesh coloration, and offer a solid foundation for breeding new apple cultivars with enhanced red pigmentation.

## 5 Methods

### 5.1 Plant Materials

In this study, *Malus* crabapple ‘Royalty’, a red fruited cultivar was used for genome sequencing. Eight‐year‐old trees were grafted onto *Malus hupehensis* and planted at the Crabapple Germplasm Resources Nursery, at the Beijing University of Agriculture (40.l° N, 116.6° E). Bagged apple (*Malus domestica cv*. ‘Red Fuji’) fruit were harvested 180 d after blooming (before full ripeness), and the bagged fruits were placed in a dark incubator for 24 h at 23°C to equilibrate the conditions. Apple calli formation was induced from young embryos of the ‘Orin’ apple cultivar ( *M. domestica* Borkh.) and calli were subcultured in MS medium (Murashig and Skoog Medium) containing 0.5 mg/L indole‐3‐acetic acid (IAA) and 1.5 mg/L 6‐benzylaminopurine (6‐BA) at 23°C in the dark. The calli were subcultured three times in 15 day intervals before being used for genetic transformation and other assays (An et al. 2020; Zhang et al. 2018). For light treatment, the harvested apples were transferred under low light from their bags to a white light incubator (390 nm‐780 nm, 12 000 lux, 23°C). Light treatment of apple calli was performed similarly. *Nicotiana benthamiana* was grown in a greenhouse at 25°C under a 16 h day, 8 h night cycle.

### 5.2 Library Construction and Sequencing

For de novo assemblies, leaves from plants of *Malus* crabapple ‘Royalty’ were collected and used for DNA extraction with the DNeasy Plant Mini Kit (QIAGEN). DNA is purified using AMPure PB beads (PacBio 100‐265‐900) to obtain high‐quality gDNA for subsequent library construction. The PacBio SMRTbell (Single‐Molecule Real‐Time) library is prepared using the SMRTbell Prep Kit 3.0. Qualified libraries are evenly loaded onto SMRT Cell chips and sequenced on the PacBio Revio platform (Pacific Biosciences, CA, USA). Using SMRT Link software to process PacBio CCS platform sequencing data to generate high‐accuracy HiFi reads.

### 5.3 Genome Size Estimation

Genome size was estimated by *k*‐mer frequency analysis. The distribution of k‐mers depends on the characteristic of the genome and follows a Poisson's distribution. Before assembly, the 17‐mer distribution of CCS reads was generated using Jellyfish (v2.2.6) (Marcais and Kingsford 2011).

### 5.4 Hi‐C Library Construction and Sequencing

DNA extraction and cross‐link was performed as previously described (Wang et al. 2023). The Hi‐C library construction and sequencing was performed as previously described (Van Berkum et al. 2010). The HiCup software (with default parameters) is used to assess the quality of HiC data, and the Valid Pairs reads are used for subsequent assembly assistance.

### 5.5 Genome Assembly and Detection of Genomic Variations

Using SMRT Link software to process PacBio CCS platform sequencing data to generate high‐accuracy HiFi reads. The HiFi data, along with Hi‐C data, are used as input files for genome assembly with Hifiasm, producing three contig sequences: Royalty_hap1, Royalty_hap2 and primary. The Hi‐C data are then aligned to the contig sequences using the HiCUP software to evaluate Hi‐C data quality. The valid reads are assessed, and the contigs are clustered, ordered, and oriented using ALLHIC software. Next, JuiceBox software is used to fine‐tune the orientation results, ultimately generating chromosome‐level genome assemblies. The assembly Royalty_hap1 and Royalty_hap2 were aligned using NUCmer (version4.4.0) (Marçais et al. 2018). We aligned the Royalty_hap1 genome to the Royalty_hap2 genome and then used SyRI (version 1.5) to identify SVs. The analysis method was as previously reported (Goel et al. 2019; Wang et al. 2023).

### 5.6 Genome Annotation

The pipeline for prediction of repeat elements included de novo and homology‐based approaches. For homologue evidence, alignment searches were undertaken against the RepBase database (http://www.girinst.org/repbase), and then were predicted by RepeatProteinMask (http://www.repeatmasker.org/). For de novo annotation, LTR_FINDER, PILER, RepeatScout (http://www.repeatmasker.org/), and Repeat‐Modeller (http://www.repeatmasker.org/RepeatModeler.html) were used to construct a *de novo* library, then annotation was carried out with Repeatmasker (http://repeatmasker.org/) (Edgar and Myers 2005; Xu and Wang 2007).

Homology‐based prediction was performed using genBlastA (http://genome.sfu.ca/projects/genBlastA/) and GeneWise (http://www.ebi.ac.uk/~birney/wise2/) (She et al. 2009). De novo predictions were obtained with Augustus (http://bioinf.uni‐greifswald.de/), GlimmerHMM (http://ccb.jhu.edu/software/glimmerhmm/), and SNAP (http://homepage.mac.com/iankorf/). The results were integrated with EVidenceModeler (EVM, http://evidencemodeler.sourceforge.net/) to generate a non‐redundant gene set, which was further refined using PASA (http://pasa.sourceforge.net/) based on transcriptome assemblies to add UTRs and alternative splicing information (Burge and Karlin 1997; Guigo 1998; Majoros et al. 2004; Korf 2004; Stanke et al. 2006). Functional annotation was carried out against protein databases including SwissProt (http://www.uniprot.org/), TrEMBL (http://www.uniprot.org/), KEGG (http://www.genome.jp/kegg/), and InterPro (https://www.ebi.ac.uk/interpro/) (Hunter et al. 2012).

### 5.7 Transcriptome Analysis

Total RNA was isolated from three biological replicates of a sampled organ at five developmental stage to investigate expression. The clean reads were mapped against the apple genome using Hisat2 (v2.0.5) software. The number of reads mapped was counted using HTSeq (v0.6.1) and then fragments per kilobase of exon per million mapped fragments values were calculated for each gene (Kim et al. 2013; Mortazavi et al. 2008). Analysis of differential gene expression between two samples was performed using the DESeq2 R package (v1.20.0) (Wang, feng, et al. 2010). Genes with an adjusted *p* value. MUSCLE (v3.8.31) was used to construct multiple nucleotide sequence align ments from the CDSs of the orthologous gene sets (Edgar 2004).

### 5.8 Kyoto Encyclopedia of Genes and Genomes (KEGG) Enrichment Analysis, Identification of Co‐Expression Modules and Visualization of Hub Genes

KOBAS software was used to test the statistical enrichment of DEGs in the KEGG pathways (http://www.genome.jp/kegg/) (Kanehisa and Goto 2000). The R WGCNA package was used to identify modules of highly correlated genes based on the fragments per kilobase of transcript per million mapped reads (FPKM) data (Langfelder and Horvath 2008). The WGCNA analysis was performed according to established methods (Yang et al. 2019). The top 150 hub genes were calculated by eigengenebased connectivity, ranked by *k* (kcor, *i*(*q*) = cor (xi, *E*(*q*))) and ME (module eigengene) (Li et al. 2020).

### 5.9 Cloning of the MdZAT5 and MdHY5 Sequences

‘Royalty’ peel cDNA was used as a template to clone the full‐length *MdZAT5* and *MdHY5* sequences. Genomic DNA was isolated from fruit peels using the Plant Genomic DNA Kit (Tiangen Biotech Co. Ltd, Beijing, China) and used as a template to amplify the promoter sequences by PCR followed by sequencing (primers listed in Table S13). *Cis*‐acting regulatory elements in the promoters were predicted using the PlantCARE database (http://bioinformatics.psb.ugent.be/webtools/plantcare/html/) (Lescot et al. 2002). Sequence comparison and analysis was performed using the advanced basic local alignment search tool (BLAST) at the National Center for Biotechnological Information (http://www.ncbi.nlm.nih.gov). The full‐length DNA and protein sequences were aligned using DNAMAN 5.2.2 (Lynnon Biosoft, USA).

### 5.10 Anthocyanin Content Determination

The anthocyanins extraction method was conducted as previously described (Tian et al. 2017). The anthocyanin level was determined by the pH differential approach (Giusti and Wrolstad 2001).

### 5.11 RNA Extraction and RT‐qPCR Analysis

Gene expression levels were analyzed by RT‐qPCR using 2×SYBR Green qPCR Mix (Vazyme, Nanjing, China) on a Bio‐Rad CFX96 Real‐Time PCR System (BIO‐RAD, Hercules, CA, USA), according to the manufacturer's instructions. RT‐qPCR analysis was carried out as described before (Ma et al. 2021). The data were analyzed using the internal control and the 2^(−∆∆Ct)^ method (Livak and Schmittgen 2001). All primer sequences are listed in Table S13. Three biological replicates of the fruit and calli samples were analyzed.

### 5.12 Construction of Expression Vectors and Stable Transformation of Apple Calli

To fuse the *MdZAT5* coding sequence with eGFP (enhanced green fluorescent protein), the corresponding cDNA were cloned into the pGFPGUSPLUS plant transformation vector downstream of the CaMV *35S* promoter (Vickers et al. 2007). The RNAi vector (pRI101‐RNAi) was made by amplifying *MdZAT5* (300 bp). All primers used are listed in Table S13. The RNAi vector (pRI101‐RNAi) was constructed by amplifying a 366 bp fragment of MdZAT5. All primers used are listed in Table S13. The construct was transformed into *Agrobacterium tumefaciens* (LBA4404) cells, which were grown, collected, and resuspended. Transformation of ‘Orin’ calli was performed as previously described (Li et al. 2012). Three independent transgenic apple callus lines were obtained for subsequent experiments.

### 5.13 Transient Expression in Apple Fruit

Bagged ‘Red Fuji’ apple fruit were harvested 170 days after blooming for transient expression. VIGS was used to silence genes in apple fruit, 366 bp conserved DNA fragments for the pTRV2‐*MdZAT5* constructs were amplified by PCR with gene‐specific primers (Table S13) from a cDNA library derived from ‘Red Fuji’ apple peel according to the manufacturer's instructions. The vectors were then used for apple fruit infiltration. *Agrobacterium tumefaciens* (GV3101) cells were grown, collected, and resuspended. The infiltration protocol and culturing methods for transient expression assays were adapted from previously described methods (Liu et al. 2002; Zhang et al. 2016). The infected apples (‘Red Fuji’) were placed at 23°C for either 5 days for overexpression and silencing treatments. Infiltration was repeated using three biological replicates.

### 5.14 Y2H, BiFC and Pull‐Down Assays

For directed Y2H assays, the MdHY5, MdbHLH3, MdJAZ2, MdCOP1 coding sequence was cloned into the pGADT7 vector as an activation domain. MdZAT5‐3G, MdZAT5‐11G^hap1^ and MdZAT5‐11G^hap2^ were cloned into the pGBKT7 vector as baits. Assays were performed according to the manufacturer's instructions (Invitrogen, Carlsbad, CA, USA). The primers used are shown in Table S13.

The in vivo BiFC assay was performed as previously described (Fu et al. 2024). MdZAT5‐3G and MdHY5 full‐length sequences were amplified and separately cloned into the pCAMBIA1300‐nLUC and pCAMBIA1300‐cLUC plasmids. MdZAT5‐3G‐GST, MdHY5‐6×His proteins were expressed in *Escherichia coli* for use in a GST pull‐down assay. The elution products were analyzed by Western blot analysis (Li et al. 2017). The primers used are shown in Table S13.

### 5.15 Y1H, Dual‐Luciferase and GUS Staining Assays

The *MdZAT5‐3G*, *MdZAT5‐11G* ^*hap1*^ and *MdZAT5‐11G* ^*hap1*^ ORFs were cloned into the *BamH* I and *Xho* I sites of the pGADT7 vector (Clontech, Palo Alto, CA, USA) under the control of the galactokinase 4 (GAL4) promoter to create the effector constructs. The *MdCHS*, *MdCHI*, *MdF3H*, *MdDFR*, *MdANS* and *MdUFGT* promoter fragments were cloned into the *EcoR* I and *Sac* I sites of the pHIS2 vector. The Y1H methods were performed as previously described (Wang et al. 2018). The primers used are shown in Table S13.

Dual‐luciferase assays were used to analyze trans‐activation of target gene promoters by TFs as previously described (Hellens et al. 2005; Xiang et al. 2015). The ORF *MdZAT5* and *MdCHS*, *MdCHI*, *MdF3H* promoter sequences were cloned into the pGreenII‐62‐SK and pGreenII‐0800‐LUC vectors, respectively, using the primers listed in Table S13 for amplification. The enzyme activity assay was carried out using three biological replicates.

*MdCHS*, *MdCHI* and *MdbF3H* promoter fragments (2000 bp) were cloned into pBI101 vector (Guerrero et al. 1990). The *MdZAT5‐3G* ORF were inserted into the pBI121 vector (Jefferson 1987). GUS staining was performed as previously described (Cai et al. 2014). *MdZAT5‐3G* ^*hap1*^, *MdZAT5‐3G* ^*hap2*^, *MdZAT5‐11G* ^*hap1*^, *MdZAT5‐11G* ^*hap2*^ promoter fragments (2000 bp) were cloned into pBI101‐GFP vector. GUS staining was performed as previously described (Ma et al. 2024). All experiments were carried out using three biological replicates.

### 5.16 Protein Structure, Binding Pocket and the Binding Pattern Prediction

Protein structure prediction using AlphaFold2 and obtaining pLDDT scores to evaluate the local structural accuracy of each residue, with higher values indicating greater confidence in the predicted structure. The protein structure prediction was performed as described in AlphaFold, and the results were scored and ranked from 0 to 5 with rank 0 representing the protein structure with the highest confidence level (over 90%); the corresponding protein structure was selected as a possible protein structure for this protein (Abramson et al. 2024). The binding pockets were predicted using DeepSite online tool (https://www.playmolecule.com/deepsite/) (Jiménez et al. 2017).

### 5.17 Statistical Analysis

Experimental data were analyzed using one‐way ANOVA followed by Tukey's multiple range test to compare differences among the experimental sites at *p* < 0.05. Student's *t*‐test, *p < 0.05; **p < 0.01. GraphPad Prism 8.0, Microsoft Excel 2016 and IBM SPSS Statistics 22 were used for analysis.

## Author Contributions

J.T. conceived and designed the research. M. Zhao, M. Zhang, W.S., and S.D. conducted the experiments. J.T. and Y.Y. contributed reagents and analytical tools. J.Z., T.S., and Y.Y. gave advice and edited the manuscript. M.Z. and J.T. wrote the manuscript. All authors read and approved the manuscript.

## Ethics Statement

The authors have nothing to report.

## Consent

The authors have nothing to report.

## Conflicts of Interest

The authors declare no conflicts of interest.

## Supporting information


**Figure S1:** Differential gene expression during fruit five development stages in fruit peel and flesh. RPs represent ‘Royalty’ peel; RFs represent ‘Royalty’ flesh.
**FIGURE S2:** Content analysis of major flavonoid compounds in ‘Royalty’ fruit at five developmental stages. (A) Major flavonoid compounds of ‘Royalty’ fruit peel. (B) Major flavonoid compounds of ‘Royalty’ fruit flesh. Error bars represent the mean ± SE of three biological replicates. Different letters above the bars indicate significant differences (*p* < 0.05), as determined by one‐way ANOVA followed by Tukey's multiple range test. Developmental stages: S1, 45 days after flowering; S2, 75 days; S3, 105 days; S4, 135 days; S5, 165 days.
**FIGURE S3:** Connectivity analysis of the two WGCNA modules, ‘MEgrey’ and ‘MEturquoise’. (A) Connectivity network analysis of ‘MEgrey’ module from ‘Royalty’ fruit peel. (B) Connectivity network analysis of ‘MEturquoise’ module from ‘Royalty’ fruit flesh.
**FIGURE S4:** Amino acid sequence alignment of MdZAT5s located on different chromosomes. (A) Sequence alignment of *MdZAT5‐3G* ^*hap1*^ and *MdZAT5‐3G* ^*hap2*^. (B) Sequence alignment of *MdZAT5‐11G* ^*hap1*^ and *MdZAT5‐11G* ^*hap2*^. (C) Sequence alignment of *MdZAT5‐3G* and *MdZAT5‐11G* ^*hap1*^. (D) Sequence alignment of *MdZAT5‐3G* and *MdZAT5‐11G* ^*hap2*^.
**FIGURE S5:** RT‐qPCR analysis of *MdZAT5‐3G* and *MdZAT5‐11G* transcript levels in apple calli following IAA treatment. The RT‐qPCR analysis were performed with three biological replicates. Error bars indicate the standard error of the mean ± SE of three replicate measurements. Different letters above the bars indicate statistically significant differences (*p* < 0.05), as determined by one‐way ANOVA followed by Tukey's multiple range test.
**FIGURE S6:** Relative expression levels of *MdMYB1* and the anthocyanin biosynthesis genes *MdDFR*, *MdANS* and *MdUFGT* in infected apple fruit as detected by RT‐qPCR. The analysis was performed using three biological replicates. Error bars represent the standard error of the mean (±SE). Different letters above the bars indicate statistically significant differences (*p* < 0.05), as determined by one‐way ANOVA followed by Tukey's multiple range test.
**FIGURE S7:** Relative expression levels of *MdMYB1* and the anthocyanin biosynthesis genes *MdDFR*, *MdANS* and *MdUFGT* in transgenic apple calli as detected by RT‐qPCR. The analysis was performed using three biological replicates. Error bars represent the standard error of the mean (±SE). Different letters above the bars indicate significantly different values (*p* < 0.05), calculated using one‐way analysis of variance (ANOVA) followed by a Tukey's multiple range test.
**FIGURE S8:** Effects of MeJA on anthocyanin accumulation in OE‐*MdZAT5‐3G* calli. (A) OE‐*MdZAT5‐3G* calli were treated with MeJA. MeJA significantly promoted anthocyanin accumulation. (B) Anthocyanin content in transgenic apple calli shown in (A). (C) Relative expression levels of *MdZAT5‐3G* and anthocyanin biosynthesis genes *MdCHS*, *MdCHI* and *MdF3H* in transgenic calli determined by RT‐qPCR. Expression levels were normalized to the empty vector or wild type (set to 1). All RT‐qPCR and anthocyanin assays were performed with three biological replicates. Error bars represent the mean ± standard error (SE) of three replicates. Different letters above bars indicate statistically significant differences (*p* < 0.05), determined by one‐way ANOVA followed by Tukey's multiple range test.
**FIGURE S9:** Relative expression levels of *MdHY5* in apple peel following light and MeJA treatment and apple calli following MeJA treatment. The RT‐qPCR analysis were performed with three biological replicates. Error bars indicate the standard error of the mean ± SE of three replicate measurements. Different letters above the bars indicate significantly different values (*p* < 0.05), calculated using one‐way analysis of variance (ANOVA) followed by a Tukey's multiple range test.
**FIGURE S10:** Predicted protein structures of MdZAT5‐3G, MdZAT5‐11G^hap1^, MdZAT5‐11G^hap2^ generated using AlphaFold. The pLDDT values indicate the local structural accuracy of each chain, with higher scores reflecting greater confidence in the predicted regions.

**Table S1:** Summary of the sequencing data for ‘Royalty’ genome assembly.
**TABLE S2:** Summary of genome assembly characteristics based on Hi‐C data.
**TABLE S3:** Summary of Hi‐C read mapping results.
**TABLE S4:** Assessment of genome assembly completeness using BUSCO.
**TABLE S5:** Assessment of genome assembly completeness using CEGMA.
**TABLE S6:** Assessment of genome assembly quality using Merqury.
**TABLE S7:** Assessment of genome assembly quality using Illumina read mapping.
**TABLE S8:** Summary of gene prediction results for the ‘Royalty’ genome.
**TABLE S9:** Summary of gene annotation results based on different databases.
**TABLE S10:** Summary of non‐coding RNA prediction.
**TABLE S11:** Statistics of chromosome assembly for the two haplotypes of the ‘Royalty’ genome.
**TABLE S12:** Statistical analysis of gene function annotation results for the ‘Royalty’ haplotype genome.
**TABLE S13:** Primers used in this study.

## Data Availability

Sequencing data of fruit peel and flesh in *Malus* Crabapple ‘Royalty’ that support the findings of this study have been deposited in the NCBI Bioproject database under accession number PRJNA1288137, PRJNA1288132, PRJNA546101 and PRJNA546107.

## References

[pbi70434-bib-0001] Abramson, J. , J. Adler , J. Dunger , et al. 2024. “Addendum: Accurate Structure Prediction of Biomolecular Interactions With AlphaFold3.” Nature 636, no. 8042: E4. 39604737 10.1038/s41586-024-08416-7 PMC11634763

[pbi70434-bib-0002] Allan, A. C. , R. P. Hellens , and W. A. Laing . 2008. “MYB Transcription Factors That Colour Our Fruit.” Trends in Plant Science 13, no. 3: 99–102. 18280199 10.1016/j.tplants.2007.11.012

[pbi70434-bib-0003] An, J. P. , F. J. Qu , J. F. Yao , et al. 2017. “The bZIP Transcription Factor MdHY5 Regulates Anthocyanin Accumulation and Nitrate Assimilation in Apple.” Horticulture Research 4: 17023. 28611922 10.1038/hortres.2017.23 PMC5461414

[pbi70434-bib-0114] An, J. P. , X. F. Wang , R. V. Espley , et al. 2020. “An Apple B‐Box Protein MdBBX37 Modulates Anthocyanin Biosynthesis and Hypocotyl Elongation Synergistically With MdMYBs and MdHY5.” Plant and Cell Physiology 61, no. 1: 2–24. 10.1093/pcp/pcz185 31550006

[pbi70434-bib-0005] An, J. P. , X. F. Wang , X. W. Zhang , S. Q. Bi , C. X. You , and Y. J. Hao . 2019. “MdBBX22 Regulates UV‐B Induced Anthocyanin Biosynthesis Through Regulating the Function of MdHY5 and Is Targeted by MdBT2 for 26S Proteasome‐Mediated Degradation.” Plant Biotechnology Journal 17, no. 12: 2231–2233. 31222855 10.1111/pbi.13196 PMC6835122

[pbi70434-bib-0006] An, J. P. , X. F. Wang , X. W. Zhang , et al. 2020. “An Apple MYB Transcription Factor Regulates Cold Tolerance and Anthocyanin Accumulation and Undergoes MIEL1‐Mediated Degradation.” Plant Biotechnology Journal 18, no. 2: 337–353. 31250952 10.1111/pbi.13201 PMC6953192

[pbi70434-bib-0007] An, J. P. , R. R. Xu , X. Liu , et al. 2021. “Jasmonate Induces Biosynthesis of Anthocyanin and Proanthocyanidin in Apple by Mediating the JAZ1‐TRB1‐MYB9 Complex.” Plant Journal 106: 1414–1430. 10.1111/tpj.15245 33759251

[pbi70434-bib-0008] An, J. P. , X. W. Zhang , C. X. You , S. Q. Bi , X. F. Wang , and Y. J. Hao . 2019. “MdWRKY40 Promotes Wounding‐Induced Anthocyanin Biosynthesis in Association With MdMYB1 and Undergoes MdBT2‐Mediated Degradation.” New Phytologist 224, no. 1: 380–395. 31225908 10.1111/nph.16008

[pbi70434-bib-0009] An, X. H. , Y. Tian , K. Q. Chen , X. F. Wang , and Y. J. Hao . 2012. “The Apple WD40 Protein MdTTG1 Interacts With bHLH but Not MYB Proteins to Regulate Anthocyanin Accumulation.” Journal of Plant Physiology 169, no. 7: 710–717. 22405592 10.1016/j.jplph.2012.01.015

[pbi70434-bib-0010] Ayvaz, H. , T. Cabaroglu , A. Akyildiz , et al. 2022. “Anthocyanins: Metabolic Digestion, Bioavailability, Therapeutic Effects, Current Pharmaceutical/Industrial Use, and Innovation Potential.” Antioxidants 12, no. 1: 48. 36670910 10.3390/antiox12010048 PMC9855055

[pbi70434-bib-0011] Ban, Y. , C. Honda , Y. Hatsuyama , M. Igarashi , H. Bessho , and T. Moriguchi . 2007. “Isolation and Functional Analysis of a MYB Transcription Factor Gene That Is a Key Regulator for the Development of Red Coloration in Apple Skin.” Plant and Cell Physiology 48, no. 7: 958–970. 17526919 10.1093/pcp/pcm066

[pbi70434-bib-0013] Boss, P. K. , C. Davies , and P. S. Robinson . 1996. “Analysis of the Expression of Anthocyanin Pathway Genes in Developing *Vitis vinifera* L. cv Shiraz Grape Berries and the Implications for Pathway Regulation.” Plant Physiology 111: 1059–1066. 12226348 10.1104/pp.111.4.1059 PMC160981

[pbi70434-bib-0014] Burge, C. , and S. Karlin . 1997. “Prediction of Complete Gene Structures in Human Genomic DNA.” Molecular Biology and Evolution 268: 78–94. 10.1006/jmbi.1997.0951 9149143

[pbi70434-bib-0015] Cai, X. T. , P. Xu , P. X. Zhao , R. Liu , L. H. Yu , and C. B. Xiang . 2014. “Arabidopsis ERF109 Mediates Cross‐Talk Between Jasmonic Acid and Auxin Biosynthesis During Lateral Root Formation.” Nature Communications 5, no. 1: 5833. 10.1038/ncomms6833 25524530

[pbi70434-bib-0016] Chagné, D. , K. Lin‐Wang , R. V. Espley , et al. 2013. “An Ancient Duplication of Apple MYB Transcription Factors is Responsible for Novel Red Fruit‐Flesh Phenotypes.” Plant Physiology 161, no. 1: 225–239. 23096157 10.1104/pp.112.206771 PMC3532254

[pbi70434-bib-0017] Chen, K. , Y. Wang , R. Zhang , H. Zhang , and C. Gao . 2019. “CRISPR/Cas Genome Editing and Precision Plant Breeding in Agriculture.” Annual Review of Plant Biology 70: 667–697. 10.1146/annurev-arplant-050718-100049 30835493

[pbi70434-bib-0018] Concha, C. M. , N. E. Figueroa , L. A. Poblete , F. A. Oñate , W. Schwab , and C. R. Figueroa . 2013. “Methyl Jasmonate Treatment Induces Changes in Fruit Ripening by Modifying the Expression of Several Ripening Genes in *Fragaria chiloensis* Fruit.” Plant Physiology and Biochemistry 70: 433–444. 23835361 10.1016/j.plaphy.2013.06.008

[pbi70434-bib-0019] Duan, N. , Y. Bai , H. Sun , et al. 2017. “Genome Re‐Sequencing Reveals the History of Apple and Supports a Two‐Stage Model for Fruit Enlargement.” Nature Communications 8, no. 1: 249. 10.1038/s41467-017-00336-7 PMC5557836 28811498

[pbi70434-bib-0020] Edgar, R. C. 2004. “MUSCLE: Multiple Sequence Alignment With High Accuracy and High Throughput.” Nucleic Acids Research 32, no. 5: 1792–1797. 15034147 10.1093/nar/gkh340 PMC390337

[pbi70434-bib-0021] Edgar, R. C. , and E. W. Myers . 2005. “PILER: Identification and Classification of Genomic Repeats.” Bioinformatics 21, no. 1: i152–i158. 15961452 10.1093/bioinformatics/bti1003

[pbi70434-bib-0022] Espley, R. V. , R. P. Hellens , J. Putterill , D. E. Stevenson , S. Kutty‐Amma , and A. C. Allan . 2007. “Red Colouration in Apple Fruit Is due to the Activity of the MYB Transcription Factor, MdMYB10.” Plant Journal 49: 414–427. 10.1111/j.1365-313X.2006.02964.x PMC1865000 17181777

[pbi70434-bib-0023] Fan, X. , J. P. Mattheis , J. K. Fellman , and M. E. Patterson . 1997. “Changes in Jasmonic Acid Concentration During Early Development of Apple Fruit.” Physiologia Plantarum 101, no. 2: 328–332.

[pbi70434-bib-0024] Fang, H. , Y. Dong , X. Yue , et al. 2019. “The B‐Box Zinc Finger Protein MdBBX20 Integrates Anthocyanin Accumulation in Response to Ultraviolet Radiation and Low Temperature.” Plant, Cell & Environment 42, no. 7: 2090–2104. 10.1111/pce.13552 30919454

[pbi70434-bib-0025] Fu, J. , L. Liao , J. Jin , et al. 2024. “A Transcriptional Cascade Involving BBX22 and HY5 Finely Regulates Both Plant Height and Fruit Pigmentation in Citrus.” Journal of Integrative Plant Biology 66, no. 8: 1752–1768. 38961693 10.1111/jipb.13719

[pbi70434-bib-0026] Gaquerel, E. , and M. Stitz . 2017. “Insect Resistance: An Emerging Molecular Framework Linking Plant Age and JA Signaling.” Molecular Plant 10, no. 4: 537–539. 28267597 10.1016/j.molp.2017.02.006

[pbi70434-bib-0028] Giusti, M. M. , and R. E. Wrolstad . 2001. “Characterization and Measurement of Anthocyanins by UV‐Visible Spectroscopy.” Current Protocols in Food Analytical Chemistry 1: F1–F2.

[pbi70434-bib-0029] Goel, M. , H. Sun , W. B. Jiao , and K. Schneeberger . 2019. “SyRI: Finding Genomic Rearrangements and Local Sequence Differences From Whole‐Genome Assemblies.” Genome Biology 20: 277. 31842948 10.1186/s13059-019-1911-0 PMC6913012

[pbi70434-bib-0030] Guerrero, F. D. , J. T. Jones , and J. E. Mullet . 1990. “Turgor‐Responsive Gene Transcription and RNA Levels Increase Rapidly When Pea Shoots Are Wilted. Sequence and Expression of Three Inducible Genes.” Plant Molecular Biology 15: 11–26. 1715781 10.1007/BF00017720

[pbi70434-bib-0031] Guigo, R. 1998. “Assembling Genes From Predicted Exons in Linear Time With Dynamic Programming.” Journal of Computational Biology 5: 681–702. 10072084 10.1089/cmb.1998.5.681

[pbi70434-bib-0034] Hellens, R. P. , A. C. Allan , E. N. Friel , et al. 2005. “Transient Expression Vectors for Functional Genomics, Quantification of Promoter Activity and RNA Silencing in Plants.” Plant Methods 1, no. 1: 1–14. 16359558 10.1186/1746-4811-1-13 PMC1334188

[pbi70434-bib-0118] Hichri, I. , L. Deluc , F. Barrieu , et al. 2011. “A Single Amino Acid Change Within the R2 Domain of the VvMYB5b Transcription Factor Modulates Affinity for Protein Partners and Target Promoters Selectivity.” BMC Plant Biology 11: 117. 21861899 10.1186/1471-2229-11-117 PMC3240579

[pbi70434-bib-0035] Honda, T. , F. Tatsuzawa , N. Kobayashi , et al. 2005. “Acylated Anthocyanins From the Violet‐Blue Flowers of *Orychophragonus violaceus* .” Phytochemistry 66, no. 15: 1844–1851. 16023157 10.1016/j.phytochem.2005.05.026

[pbi70434-bib-0036] Huang, D. , L. Xue , Y. Lu , et al. 2024. “PpBBX32 and PpZAT5 Modulate Temperature‐Dependent and Tissue‐Specific Anthocyanin Accumulation in Peach Fruit.” Horticulture Research 11, no. 10: uhae212. 39385999 10.1093/hr/uhae212 PMC11462610

[pbi70434-bib-0037] Hunter, S. , P. Jones , A. Mitchell , et al. 2012. “InterPro in 2011: New Developments in the Family and Domain Prediction Database.” Nucleic Acids Research 40: D306–D312. 22096229 10.1093/nar/gkr948 PMC3245097

[pbi70434-bib-0038] Ijinu, T. P. , L. F. De Lellis , S. Shanmugarama , et al. 2023. “Anthocyanins as Immunomodulatory Dietary Supplements: A Nutraceutical Perspective and Micro‐/Nano‐Strategies for Enhanced Bioavailability.” Nutrients 15, no. 19: 4152. 37836436 10.3390/nu15194152 PMC10574533

[pbi70434-bib-0116] Jefferson, R. A. , T. A. Kavanagh , and M. W. Bevan . 1987. “GUS Fusions: Beta‐Glucuronidase as a Sensitive and Versatile Gene Fusion Marker in Higher Plants.” EMBO Journal 6, no. 13: 3901–3907. 3327686 10.1002/j.1460-2075.1987.tb02730.x PMC553867

[pbi70434-bib-0039] Jiménez, J. , S. Doerr , G. Martínez‐Rosell , A. S. Rose , and G. De Fabritiis . 2017. “DeepSite: Protein‐Binding Site Predictor Using 3D‐Convolutional Neural Networks.” Bioinformatics (Oxford, England) 33, no. 19: 3036–3042. 28575181 10.1093/bioinformatics/btx350

[pbi70434-bib-0040] Kanehisa, M. , and S. Goto . 2000. “KEGG: Kyoto Encyclopedia of Genes and Genomes.” Nucleic Acids Research 28, no. 1: 27–30. 10592173 10.1093/nar/28.1.27 PMC102409

[pbi70434-bib-0115] Khan, A. , S. B. Carey , A. Serrano , et al. 2022. “A Phased, Chromosome‐Scale Genome of ‘Honeycrisp’ Apple (*Malus domestica*).” GigaByte: gigabyte69. 36824509 10.46471/gigabyte.69 PMC9693968

[pbi70434-bib-0041] Kiełbowicz‐Matuk, A. 2012. “Involvement of Plant C2H2‐Type Zinc Finger Transcription Factors in Stress Responses.” Plant Science 185: 78–85. 22325868 10.1016/j.plantsci.2011.11.015

[pbi70434-bib-0042] Kim, D. , G. Pertea , C. Trapnell , H. Pimentel , R. Kelley , and S. L. Salzberg . 2013. “TopHat2: Accurate Alignment of Transcriptomes in the Presence of Insertions, Deletions and Gene Fusions.” Genome Biology 14: R36. 23618408 10.1186/gb-2013-14-4-r36 PMC4053844

[pbi70434-bib-0043] Konczak, I. , and W. Zhang . 2004. “Anthocyanins‐More Than Nature's Colours.” Journal of Biomedicine and Biotechnology 5: 239. 10.1155/S1110724304407013 PMC1082903 15577183

[pbi70434-bib-0044] Korf, I. 2004. “Gene Finding in Novel Genomes.” BMC Bioinformatics 5: 59. 15144565 10.1186/1471-2105-5-59 PMC421630

[pbi70434-bib-0045] Langfelder, P. , and S. Horvath . 2008. “WGCNA: An R Package for Weighted Correlation Network Analysis.” BMC Bioinformatics 9: 1–13. 19114008 10.1186/1471-2105-9-559 PMC2631488

[pbi70434-bib-0046] Lescot, M. , P. Déhais , G. Thijs , et al. 2002. “PlantCARE, a Database of Plant Cis‐Acting Regulatory Elements and a Portal to Tools for In Silico Analysis of Promoter Sequences.” Nucleic Acids Research 30, no. 1: 325–327. 11752327 10.1093/nar/30.1.325 PMC99092

[pbi70434-bib-0047] Li, H. , Y. Li , J. Yu , et al. 2020. “MdMYB8 Is Associated With Flavonol Biosynthesis via the Activation of the *MdFLS* Promoter in the Fruits of *Malus* Crabapple.” Horticulture Research 7: 19. 32025322 10.1038/s41438-020-0238-z PMC6994661

[pbi70434-bib-0048] Li, T. , Y. Xu , L. Zhang , et al. 2017. “The Jasmonate‐Activated Transcription Factor MdMYC2 Regulates ETHYLENE RESPONSE FACTOR and Ethylene Biosynthetic Genes to Promote Ethylene Biosynthesis During Apple Fruit Ripening.” Plant Cell 29: 1316–1334. 28550149 10.1105/tpc.17.00349 PMC5502464

[pbi70434-bib-0049] Li, Y. , P. Li , L. Zhang , et al. 2022. “Genome‐Wide Analysis of the Apple Family I Glycosyltransferases Identified a Flavonoid‐Modifying UGT, MdUGT83L3, Which Is Targeted by MdMYB88 and Contributes to Stress Adaptation.” Plant Science 321: 111314. 35696914 10.1016/j.plantsci.2022.111314

[pbi70434-bib-0050] Li, Y. Y. , K. Mao , C. Zhao , et al. 2012. “MdCOP1 Ubiquitin E3 Ligases Interact With MdMYB1 to Regulate Light‐Induced Anthocyanin Biosynthesis and Red Fruit Coloration in Apple.” Plant Physiology 160, no. 2: 1011–1022. 22855936 10.1104/pp.112.199703 PMC3461526

[pbi70434-bib-0051] Liu, H. , B. Reavy , M. Swanson , and S. A. MacFarlane . 2002. “Functional Replacement of the Tobacco Rattle Virus Cysteine‐Rich Protein by Pathogenicity Proteins From Unrelated Plant Viruses.” Virology 298, no. 2: 232–239. 12127786 10.1006/viro.2002.1421

[pbi70434-bib-0052] Liu, W. , Z. Mei , L. Yu , et al. 2023. “The ABA‐Induced NAC Transcription Factor MdNAC1 Interacts With a bZIP‐Type Transcription Factor to Promote Anthocyanin Synthesis in Red‐Fleshed Apples.” Horticulture Research 10, no. 5: uhad049. 37200839 10.1093/hr/uhad049 PMC10186271

[pbi70434-bib-0053] Livak, K. J. , and T. D. Schmittgen . 2001. “Analysis of Relative Gene Expression Data Using Real‐Time Quantitative PCR and the 2^−ΔΔCT^ Method.” Methods 25, no. 4: 402–408. 11846609 10.1006/meth.2001.1262

[pbi70434-bib-0117] Ma, H. , M. Fu , Z. Xu , et al. 2024. “Allele‐Specific Expression of AP2‐Like ABA Repressor 1 Regulates Iron Uptake by Modulating Rhizosphere pH in Apple.” Plant Physiology 196, no. 3: 2121–2136. 39197038 10.1093/plphys/kiae452

[pbi70434-bib-0054] Ma, H. Y. , T. Yang , Y. Li , et al. 2021. “The Long Noncoding RNA MdLNC499 Bridges MdWRKY1 and MdERF109 Function to Regulate Early‐Stage Light‐Induced Anthocyanin Accumulation in Apple Fruit.” Plant Cell 33, no. 10: 3309–3330. 34270784 10.1093/plcell/koab188 PMC8505877

[pbi70434-bib-0055] Majoros, W. H. , M. Pertea , and S. L. Salzberg . 2004. “TigrScan and GlimmerHMM: Two Open Source Ab Initio Eukaryotic Gene‐Finders.” Bioinformatics 20: 2878–2879. 15145805 10.1093/bioinformatics/bth315

[pbi70434-bib-0057] Marcais, G. , and C. Kingsford . 2011. “A Fast, Lock‐Free Approach for Efficient Parallel Counting of Occurrences of k‐mers.” Bioinformatics 27, no. 6: 764–770. 21217122 10.1093/bioinformatics/btr011 PMC3051319

[pbi70434-bib-0056] Marçais, G. , A. L. Delcher , A. M. Phillippy , R. Coston , S. L. Salzberg , and A. Zimin . 2018. “MUMmer4: A Fast and Versatile Genome Alignment System.” PLoS Computational Biology 14, no. 1: e1005944. 29373581 10.1371/journal.pcbi.1005944 PMC5802927

[pbi70434-bib-0058] Meng, J. , S. Sun , A. Li , et al. 2023. “A NAC Transcription Factor, PpNAC1, Regulates the Expression of PpMYB10.1 to Promote Anthocyanin Biosynthesis in the Leaves of Peach Trees in Autumn.” Horticulture Advances 1, no. 1: 8.

[pbi70434-bib-0059] Mortazavi, A. , B. A. Williams , K. McCue , L. Schaeffer , and B. Wold . 2008. “Mapping and Quantifying Mammalian Transcriptomes by RNA‐Seq.” Nature Methods 5, no. 7: 621–628. 18516045 10.1038/nmeth.1226 PMC13303166

[pbi70434-bib-0060] Oravecz, A. , A. Baumann , Z. Máté , et al. 2006. “Constitutively Photomorphogenic1 Is Required for the UV‐B Response in *Arabidopsis* .” Plant Cell 18, no. 8: 1975–1990. 16829591 10.1105/tpc.105.040097 PMC1533968

[pbi70434-bib-0061] Ortigosa, A. , S. Fonseca , J. M. Franco‐Zorrilla , et al. 2020. “The JA‐Pathway MYC Transcription Factors Regulate Photomorphogenic Responses by Targeting HY5 Gene Expression.” Plant Journal 102, no. 1: 138–152. 10.1111/tpj.14618 31755159

[pbi70434-bib-0062] Osterlund, M. T. , C. S. Hardtke , N. Wei , and X. W. Deng . 2000. “Targeted Destabilization of HY5 During Light‐Regulated Development of Arabidopsis.” Nature 405, no. 6785: 462–466. 10839542 10.1038/35013076

[pbi70434-bib-0063] Parra, G. , K. Bradnam , and I. Korf . 2007. “CEGMA: A Pipeline to Accurately Annotate Core Genes in Eukaryotic Genomes.” Bioinformatics 23, no. 9: 1061–1067. 17332020 10.1093/bioinformatics/btm071

[pbi70434-bib-0064] Prasad, B. R. , S. V. Kumar , A. Nandi , and S. Chattopadhyay . 2012. “Functional Interconnections of HY1 With MYC2 and HY5 in *Arabidopsis* Seedling Development.” BMC Plant Biology 12: 37. 22424472 10.1186/1471-2229-12-37 PMC3353174

[pbi70434-bib-0065] Putterill, J. , F. Robson , K. Lee , R. Simon , and G. Coupland . 1995. “The CONSTANS Gene of *Arabidopsis* Promotes Flowering and Encodes a Protein Showing Similarities to Zinc Finger Transcription Factors.” Cell 80, no. 6: 847–857. 7697715 10.1016/0092-8674(95)90288-0

[pbi70434-bib-0066] Ramsay, N. A. , and B. J. Glover . 2005. “MYB‐bHLH‐WD40 Protein Complex and the Evolution of Cellular Diversity.” Trends in Plant Science 10, no. 2: 63–70. 15708343 10.1016/j.tplants.2004.12.011

[pbi70434-bib-0067] Rhie, A. , B. P. Walenz , S. Koren , and A. M. Phillippy . 2020. “Merqury: Reference‐Free Quality, Completeness, and Phasing Assessment for Genome Assemblies.” Genome Biology 21, no. 1: 1–27. 10.1186/s13059-020-02134-9 PMC7488777 32928274

[pbi70434-bib-0068] Saniewski, M. , J. Nowacki , and J. Czapski . 1987. “The Effect of Methyl Jasmonate on Ethylene Production and Ethylene‐Forming Enzyme Activity in Tomatoes.” Journal of Plant Physiology 129, no. 1–2: 175–180.

[pbi70434-bib-0069] Seppey, M. , M. Manni , and E. M. Zdobnov . 2019. “BUSCO: Assessing Genome Assembly and Annotation Completeness.” Gene Prediction: Methods and Protocols 1962: 227–245. 10.1007/978-1-4939-9173-0_14 31020564

[pbi70434-bib-0070] She, R. , J. S. Chu , K. Wang , J. Pei , and N. Chen . 2009. “genBlastA: Enabling BLAST to Identify Homologous Gene Sequences.” Genome Research 19: 143–149. 18838612 10.1101/gr.082081.108 PMC2612959

[pbi70434-bib-0071] Shi, H. , G. Liu , Y. Wei , and Z. Chan . 2018. “The Zinc‐Finger Transcription Factor ZAT6 Is Essential for Hydrogen Peroxide Induction of Anthocyanin Synthesis in *Arabidopsis* .” Plant Molecular Biology 97, no. 1–2: 165–176. 29675814 10.1007/s11103-018-0730-0

[pbi70434-bib-0072] Soltis, D. E. , V. A. Albert , J. Leebens‐Mack , et al. 2009. “Polyploidy and Angiosperm Diversification.” American Journal of Botany 96, no. 1: 336–348. 21628192 10.3732/ajb.0800079

[pbi70434-bib-0073] Stanke, M. , O. Keller , I. Gunduz , A. Hayes , S. Waack , and B. Morgenstern . 2006. “AUGUSTUS: Ab Initio Prediction of Alternative Transcripts.” Nucleic Acids Research 34: W435–W439. 16845043 10.1093/nar/gkl200 PMC1538822

[pbi70434-bib-0074] Sun, Q. G. , S. H. Jiang , T. L. Zhang , et al. 2019. “Apple NAC Transcription Factor MdNAC52 Regulates Biosynthesis of Anthocyanin and Proanthocyanidin Through MdMYB9 and MdMYB11.” Plant Science 289: 110286. 31623786 10.1016/j.plantsci.2019.110286

[pbi70434-bib-0075] Takos, A. M. , F. W. Jaffé , S. R. Jacob , J. Bogs , S. P. Robinson , and A. R. Walker . 2006. “Light‐Induced Expression of a MYB Gene Regulates Anthocyanin Biosynthesis in Red Apples.” Plant Physiology 142, no. 3: 1216–1232. 17012405 10.1104/pp.106.088104 PMC1630764

[pbi70434-bib-0113] Tanaka, Y. , N. Sasaki , and A. Ohmiya . 2008. “Biosynthesis of Plant Pigments: Anthocyanins, Betalains and Carotenoids.” Plant Journal 54, no. 4: 733–749. 10.1111/j.1365-313X.2008.03447.x 18476875

[pbi70434-bib-0076] Tian, J. , Z. Y. Han , L. R. Zhang , et al. 2015. “Induction of Anthocyanin Accumulation in Crabapple (*Malus* cv.) Leaves by Low Temperatures.” HortScience 50, no. 5: 640–649.

[pbi70434-bib-0077] Tian, J. , J. Zhang , Z. Y. Han , et al. 2017. “McMYB12 Transcription Factors Co‐Regulate Proanthocyanidin and Anthocyanin Biosynthesis in *Malus* Crabapple.” Scientific Reports 7, no. 1: 43715. 28255171 10.1038/srep43715 PMC5334656

[pbi70434-bib-0078] Upadhyay, N. , and N. Gupta . 2024. “Diagnosis of Fungi Affected Apple Crop Disease Using Improved ResNeXt Deep Learning Model.” Multimedia Tools and Applications 83, no. 24: 64879–64898.

[pbi70434-bib-0079] Vaknin, H. , A. Bar‐Akiva , and R. Ovadia . 2005. “Active Anthocyanin Degradation in *Brunfelsia calycina* (Yesterday‐Today‐Tomorrow) Flowers.” Planta 222: 19–26. 15918029 10.1007/s00425-005-1509-5

[pbi70434-bib-0080] Van Berkum, N. L. , E. Lieberman‐Aiden , L. Williams , et al. 2010. “Hi‐C: A Method to Study the Three‐Dimensional Architecture of Genomes.” Journal of Visualized Experiments: JoVE 39: 1869. 10.3791/1869 PMC3149993 20461051

[pbi70434-bib-0081] Velasco, R. , A. Zharkikh , J. Affourtit , et al. 2010. “The Genome of the Domesticated Apple (*Malus domestica* Borkh.).” Nature Genetics 42, no. 10: 833–839. 20802477 10.1038/ng.654

[pbi70434-bib-0082] Vickers, C. E. , P. M. Schenk , D. Li , P. M. Mullineaux , and P. M. Gresshoff . 2007. “pGFPGUS Plus, a New Binary Vector for Gene Expression Studies and Optimising Transformation Systems in Plants.” Biotechnology Letters 29: 1793–1796. 17687623 10.1007/s10529-007-9467-6

[pbi70434-bib-0084] Wang, L. , Z. Feng , X. Wang , X. Wang , and X. Zhang . 2010. “DEGseq: An R Package for Identifying Differentially Expressed Genes From RNA‐Seq Data.” Bioinformatics 26, no. 1: 136–138. 19855105 10.1093/bioinformatics/btp612

[pbi70434-bib-0085] Wang, N. , W. Liu , Z. Mei , et al. 2024. “A Functional inDel in the WRKY10 Promoter Controls the Degree of Flesh Red Pigmentation in Apple.” Advanced Science 11, no. 30: e2400998. 38874015 10.1002/advs.202400998 PMC11321683

[pbi70434-bib-0086] Wang, T. , S. Duan , C. Xu , et al. 2023. “Pan‐Genome Analysis of 13 *Malus* Accessions Reveals Structural and Sequence Variations Associated With Fruit Traits.” Nature Communications 14, no. 1: 7377. 10.1038/s41467-023-43270-7 PMC10651928 37968318

[pbi70434-bib-0087] Wang, Y. , W. Liu , H. Jiang , et al. 2019. “The R2R3‐MYB Transcription Factor MdMYB24‐Like Is Involved in Methyl Jasmonate‐Induced Anthocyanin Biosynthesis in Apple.” Plant Physiology and Biochemistry 139: 273–282. 30925437 10.1016/j.plaphy.2019.03.031

[pbi70434-bib-0119] Wang, Y. C. , N. Wang , H. F. Xu , et al. 2018. “Auxin Regulates Anthocyanin Biosynthesis Through the Aux/IAA‐ARF Signaling Pathway in Apple.” Horticulture Research 5: 59. 30534386 10.1038/s41438-018-0068-4 PMC6269505

[pbi70434-bib-0088] Wang, Y. , Z. Zhai , Y. Sun , et al. 2021. “Genome‐Wide Identification of the B‐Box Genes That Respond to Multiple Ripening Related Signals in Sweet Cherry Fruit.” International Journal of Molecular Sciences 22, no. 4: 1622. 33562756 10.3390/ijms22041622 PMC7914455

[pbi70434-bib-0089] Wang, Y. , J. Zhang , and L. Zhang . 2022. “An Active and pH‐Responsive Film Developed by Sodium Carboxymethyl Cellulose/Polyvinyl Alcohol Doped With Rose Anthocyanin Extracts.” Food Chemistry 373: 131367. 34731797 10.1016/j.foodchem.2021.131367

[pbi70434-bib-0090] Wang, Z. , H. Du , R. Zhai , L. Song , F. Ma , and L. Xu . 2017. “Transcriptome Analysis Reveals Candidate Genes Related to Color Fading of ‘Red Bartlett’ (*Pyrus communis* L.).” Frontiers in Plant Science 8: 455. 28408914 10.3389/fpls.2017.00455 PMC5374147

[pbi70434-bib-0091] Wasternack, C. , and B. Hause . 2013. “Jasmonates: Biosynthesis, Perception, Signal Transduction and Action in Plant Stress Response, Growth and Development. An Update to the 2007 Review in *Annals of Botany* .” Annals of Botany 111, no. 6: 1021–1058. 23558912 10.1093/aob/mct067 PMC3662512

[pbi70434-bib-0092] Weischenfeldt, J. , O. Symmons , F. Spitz , and J. O. Korbel . 2013. “Phenotypic Impact of Genomic Structural Variation: Insights From and for Human Disease.” Nature Reviews. Genetics 14, no. 2: 125–138. 10.1038/nrg3373 23329113

[pbi70434-bib-0093] Winkel‐Shirley, B. 2001. “Flavonoid Biosynthesis. A Colorful Model for Genetics, Biochemistry, Cell Biology, and Biotechnology.” Plant Physiology 126, no. 2: 485–493. 11402179 10.1104/pp.126.2.485 PMC1540115

[pbi70434-bib-0094] Xiang, L. L. , X. F. Liu , X. Li , et al. 2015. “A Novel bHLH Transcription Factor Involved in Regulating Anthocyanin Biosynthesis in Chrysanthemums ( *Chrysanthemum morifolium* Ramat.).” PLoS One 10, no. 11: e0143892. 26619181 10.1371/journal.pone.0143892 PMC4664390

[pbi70434-bib-0095] Xing, Y. , W. Sun , Y. Sun , et al. 2023. “MPK6‐Mediated HY5 Phosphorylation Regulates Light‐Induced Anthocyanin Accumulation in Apple Fruit.” Plant Biotechnology Journal 21, no. 2: 283–301. 36208018 10.1111/pbi.13941 PMC9884024

[pbi70434-bib-0096] Xu, Z. , and H. Wang . 2007. “LTR_FINDER: An Efficient Tool for the Prediction of Full‐Length LTR Retrotransposons.” Nucleic Acids Research 35: W265–W268. 17485477 10.1093/nar/gkm286 PMC1933203

[pbi70434-bib-0097] Yang, L. , X. Zhou , Y. Deng , D. Gong , H. Luo , and P. Zhu . 2021. “Dissipation Behavior, Residue Distribution, and Dietary Risk Assessment of Fluopimomide and Dimethomorph in Taro Using HPLC‐MS/MS.” Environmental Science and Pollution Research 28: 43956–43969. 33846922 10.1007/s11356-021-13713-z

[pbi70434-bib-0098] Yang, T. , H. Ma , J. Zhang , et al. 2019. “Systematic Identification of Long Noncoding RNAs Expressed During Light‐Induced Anthocyanin Accumulation in Apple Fruit.” Plant Journal 100, no. 3: 572–590. 10.1111/tpj.14470 31344284

[pbi70434-bib-0100] Yu, X. J. , H. K. Zheng , J. Wang , W. Wang , and B. Su . 2006. “Detecting Lineage‐Specific Adaptive Evolution of Brain‐Expressed Genes in Human Using Rhesus Macaque as Outgroup.” Genomics 88: 745–751. 16857340 10.1016/j.ygeno.2006.05.008

[pbi70434-bib-0101] Zhang, J. , J. Tian , D. Q. Tai , K. T. Li , Y. J. Zhu , and Y. C. Yao . 2016. “An Optimized TRV‐Based Virus‐Induced Gene Silencing Protocol for *Malus crabapple* .” Plant Cell, Tissue and Organ Culture 126: 499–509.

[pbi70434-bib-0102] Zhang, J. , H. Xu , N. Wang , et al. 2018. “The Ethylene Response Factor MdERF1B Regulates Anthocyanin and Proanthocyanidin Biosynthesis in Apple.” Plant Molecular Biology 98, no. 3: 205–218. 30182194 10.1007/s11103-018-0770-5

[pbi70434-bib-0103] Zhang, L. , J. Hu , X. Han , et al. 2019. “A High‐Quality Apple Genome Assembly Reveals the Association of a Retrotransposon and Red Fruit Colour.” Nature Communications 10, no. 1: 1494. 10.1038/s41467-019-09518-x PMC6445120 30940818

[pbi70434-bib-0104] Zhang, L. , R. Tao , S. Wang , et al. 2022. “PpZAT5 Suppresses the Expression of a B‐Box Gene PpBBX18 to Inhibit Anthocyanin Biosynthesis in the Fruit Peel of Red Pear.” Frontiers in Plant Science 13: 1022034. 36304405 10.3389/fpls.2022.1022034 PMC9592862

[pbi70434-bib-0105] Zhou, C. , C. Zhu , H. Fu , et al. 2019. “Genome‐Wide Investigation of Superoxide Dismutase (SOD) Gene Family and Their Regulatory miRNAs Reveal the Involvement in Abiotic Stress and Hormone Response in Tea Plant ( *Camellia sinensis* ).” PLoS One 14, no. 10: e0223609. 31600284 10.1371/journal.pone.0223609 PMC6786557

