# Supplementary material for: Genome Assembly Revealed MdZAT5 Coordinates Anthocyanin Biosynthesis in Apple Fruit Peel and Flesh by Interacting With MdHY5
Source: Plant Biotechnol J. 2025 Nov 3;24(3):1655–77. doi: 10.1111/pbi.70434 (PMC12946501; doi:10.1111/pbi.70434)
Supplement: Supplementary file 1 — Figure S1: Differential gene expression during fruit five development stages in fruit peel and flesh. RPs represent ‘Royalty’ peel; RFs represent ‘Royalty’ flesh. FIGURE S2: Content analysis of major flavonoid compounds in ‘Royalty’ fruit at five developmental stages. (A) Major flavonoid compounds of ‘Royalty’ fruit peel. (B) Major flavonoid compounds of ‘Royalty’ fruit flesh. Error bars represent the mean ± SE of three biological replicates. Different letters above the bars indicate significant differences (p < 0.05), as determined by one‐way ANOVA followed by Tukey's multiple range test. Developmental stages: S1, 45 days after flowering; S2, 75 days; S3, 105 days; S4, 135 days; S5, 165 days. FIGURE S3: Connectivity analysis of the two WGCNA modules, ‘MEgrey’ and ‘MEturquoise’. (A) Connectivity network analysis of ‘MEgrey’ module from ‘Royalty’ fruit peel. (B) Connectivity network analysis of ‘MEturquoise’ module from ‘Royalty’ fruit flesh. FIGURE S4: Amino acid sequence alignment of MdZAT5s located on different chromosomes. (A) Sequence alignment of MdZAT5‐3G hap1 and MdZAT5‐3G hap2 . (B) Sequence alignment of MdZAT5‐11G hap1 and MdZAT5‐11G hap2 . (C) Sequence alignment of MdZAT5‐3G and MdZAT5‐11G hap1 . (D) Sequence alignment of MdZAT5‐3G and MdZAT5‐11G hap2 . FIGURE S5: RT‐qPCR analysis of MdZAT5‐3G and MdZAT5‐11G transcript levels in apple calli following IAA treatment. The RT‐qPCR analysis were performed with three biological replicates. Error bars indicate the standard error of the mean ± SE of three replicate measurements. Different letters above the bars indicate statistically significant differences (p < 0.05), as determined by one‐way ANOVA followed by Tukey's multiple range test. FIGURE S6: Relative expression levels of MdMYB1 and the anthocyanin biosynthesis genes MdDFR, MdANS and MdUFGT in infected apple fruit as detected by RT‐qPCR. The analysis was performed using three biological replicates. Error bars represent the standard error of the mean (±SE). [file PBI-24-1655-s001.docx]

**Supplemental Figures**


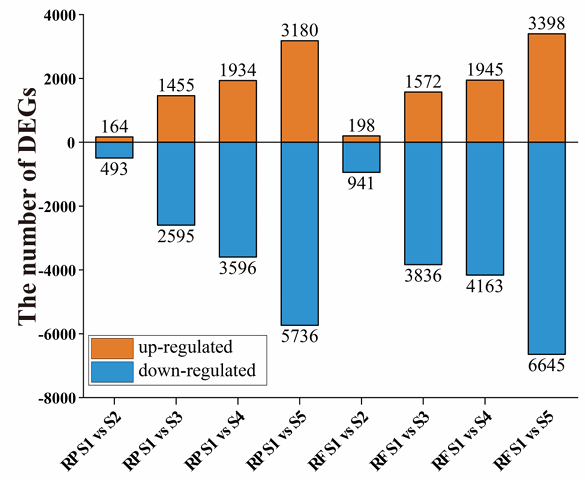


**Figure S1.** Differential gene expression during fruit five development stages in fruit peel and flesh. RPs represent ‘Royalty’ peel; RFs represent ‘Royalty’ flesh.


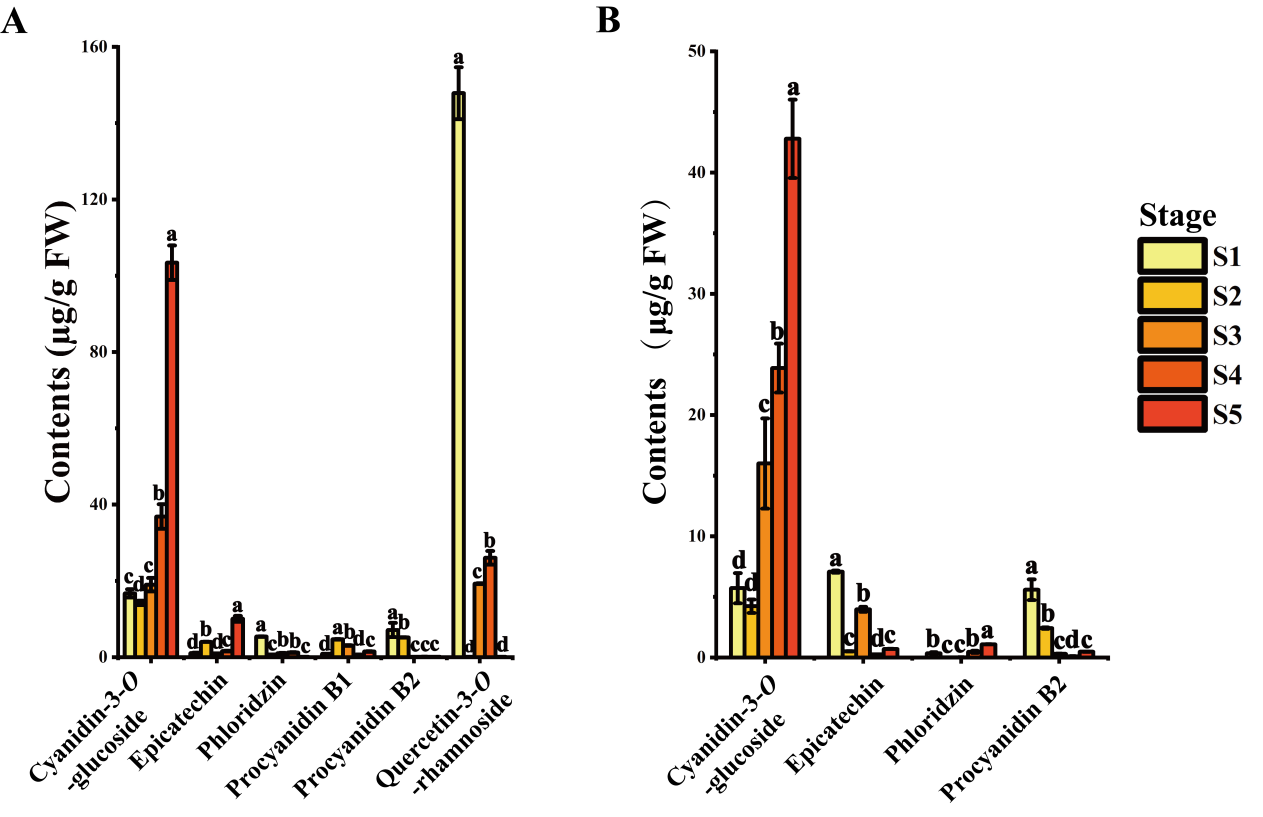


**Figure S2.** Content analysis of major flavonoid compounds in ‘Royalty’ fruit at five developmental stages. (A) Major flavonoid compounds of ‘Royalty’ fruit peel. (B) Major flavonoid compounds of ‘Royalty’ fruit flesh. Error bars represent the mean ± SE of three biological replicates. Different letters above the bars indicate significant differences (*P* < 0.05), as determined by one-way ANOVA followed by Tukey’s multiple range test. Developmental stages: S1, 45 days after flowering; S2, 75 days; S3, 105 days; S4, 135 days; S5, 165 days.


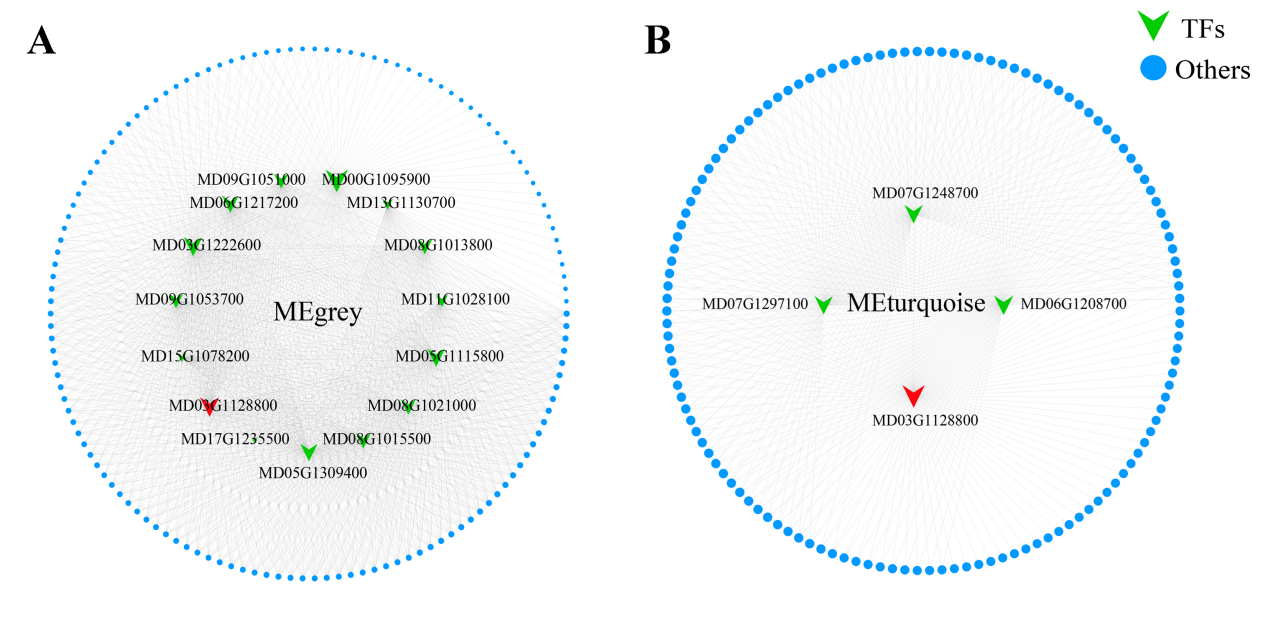


**Figure S3.** Connectivity analysis of the two WGCNA modules, ‘MEgrey’ and ‘MEturquoise’. (A) Connectivity network analysis of ‘MEgrey’ module from ‘Royalty’ fruit peel. (B) Connectivity network analysis of ‘MEturquoise’ module from ‘Royalty’ fruit flesh.


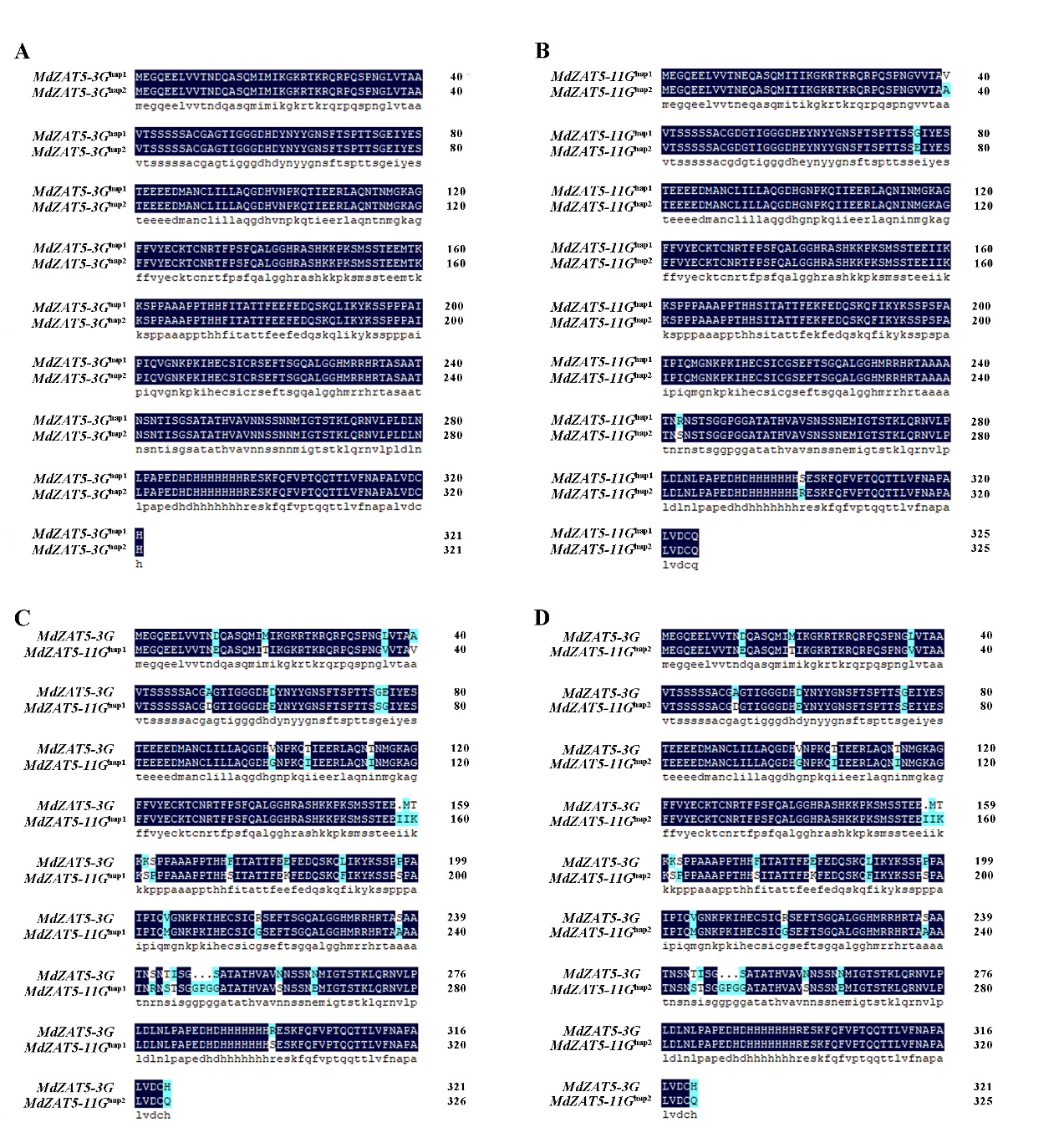


**Figure S4.** Amino acid sequence alignment of MdZAT5 in different chromosomes. (A) Sequence alignment of *MdZAT5-3G^hap1^* with *MdZAT5-3G^hap2^*. (B) Sequence alignment of *MdZAT5-11G^hap1^* with *MdZAT5-11G^hap2^*. (C) Sequence alignment of *MdZAT5-3G* with *MdZAT5-11G^hap1^*. (D) Sequence alignment of *MdZAT5-3G* with *MdZAT5-11G^hap2^.*


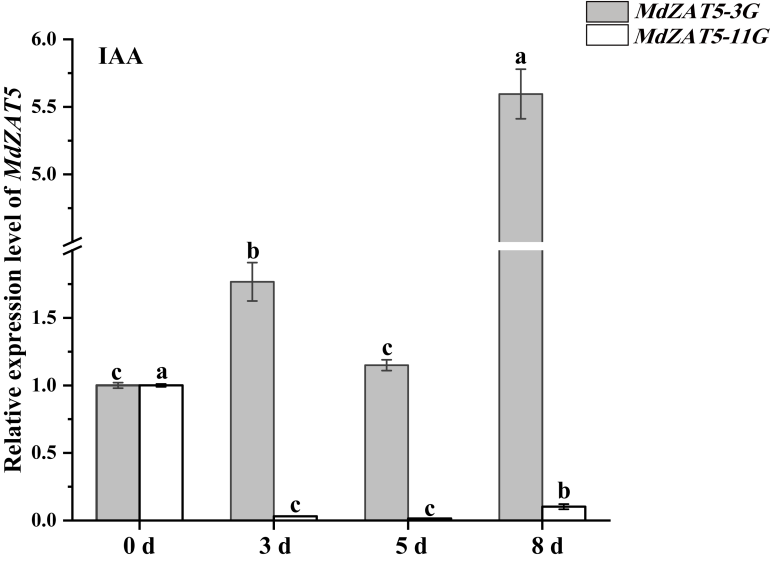


**Figure S5.** RT-qPCR analysis of *MdZAT5-3G* and *MdZAT5-11G* transcript levels in apple calli following IAA treatment. The RT-qPCR analysis were performed with three biological replicates. Error bars indicate the standard error of the mean ± SE of three replicate measurements. Different letters above the bars indicate significantly different values (*P* < 0.05), calculated using one-way analysis of variance (ANOVA) followed by a Tukey’s multiple range test.

**
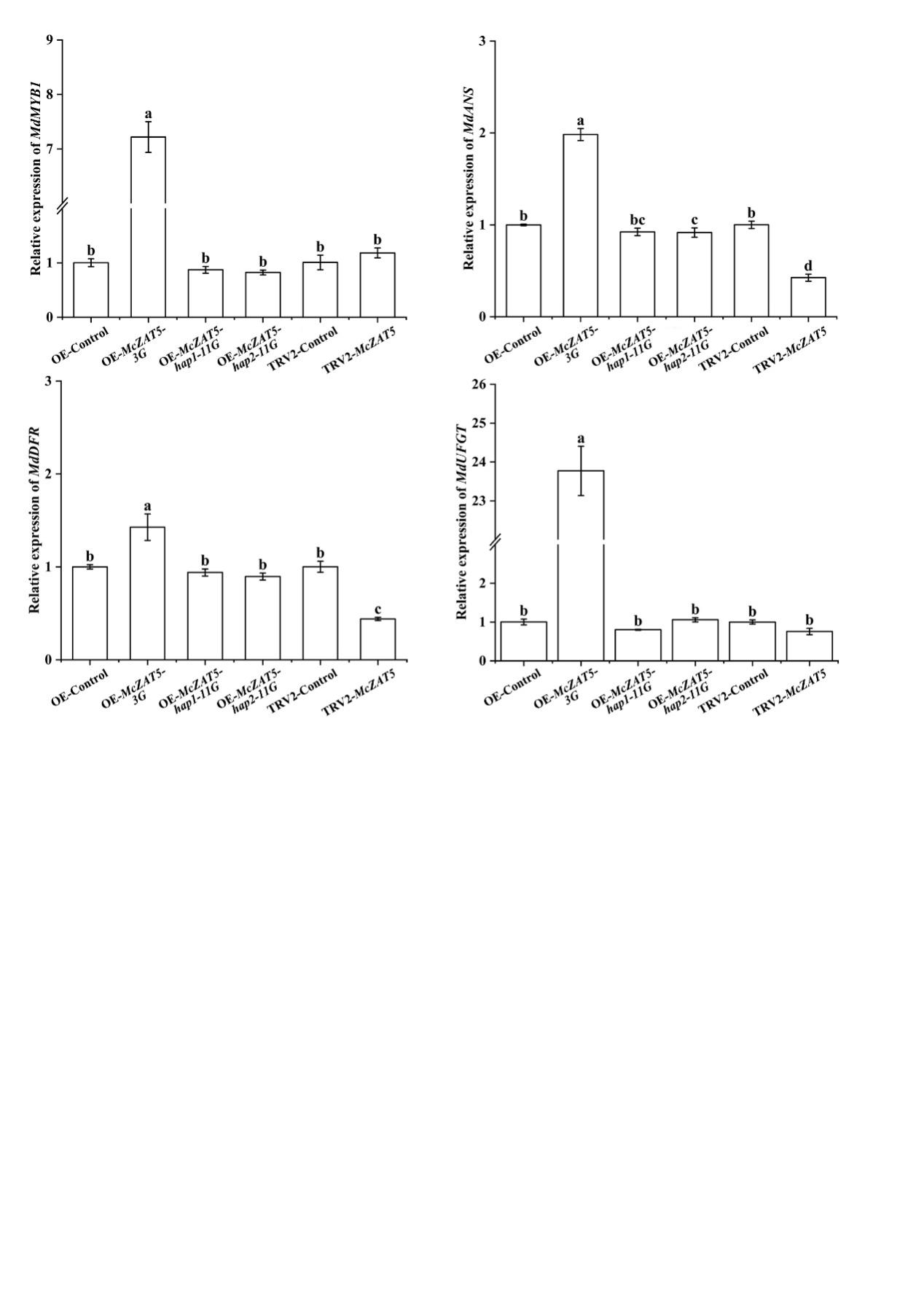
Figure S6.** Relative expression levels of *MdMYB1* and the anthocyanin biosynthesis genes *MdDFRS*, *MdANS* and *MdUFGT* in inoculated apple fruit as detected by RT-qPCR. The RT-qPCR analysis were performed with three biological replicates. Error bars indicate the standard error of the mean ± SE of three replicate measurements. Different letters above the bars indicate significantly different values (*P* < 0.05), calculated using one-way analysis of variance (ANOVA) followed by a Tukey’s multiple range test.


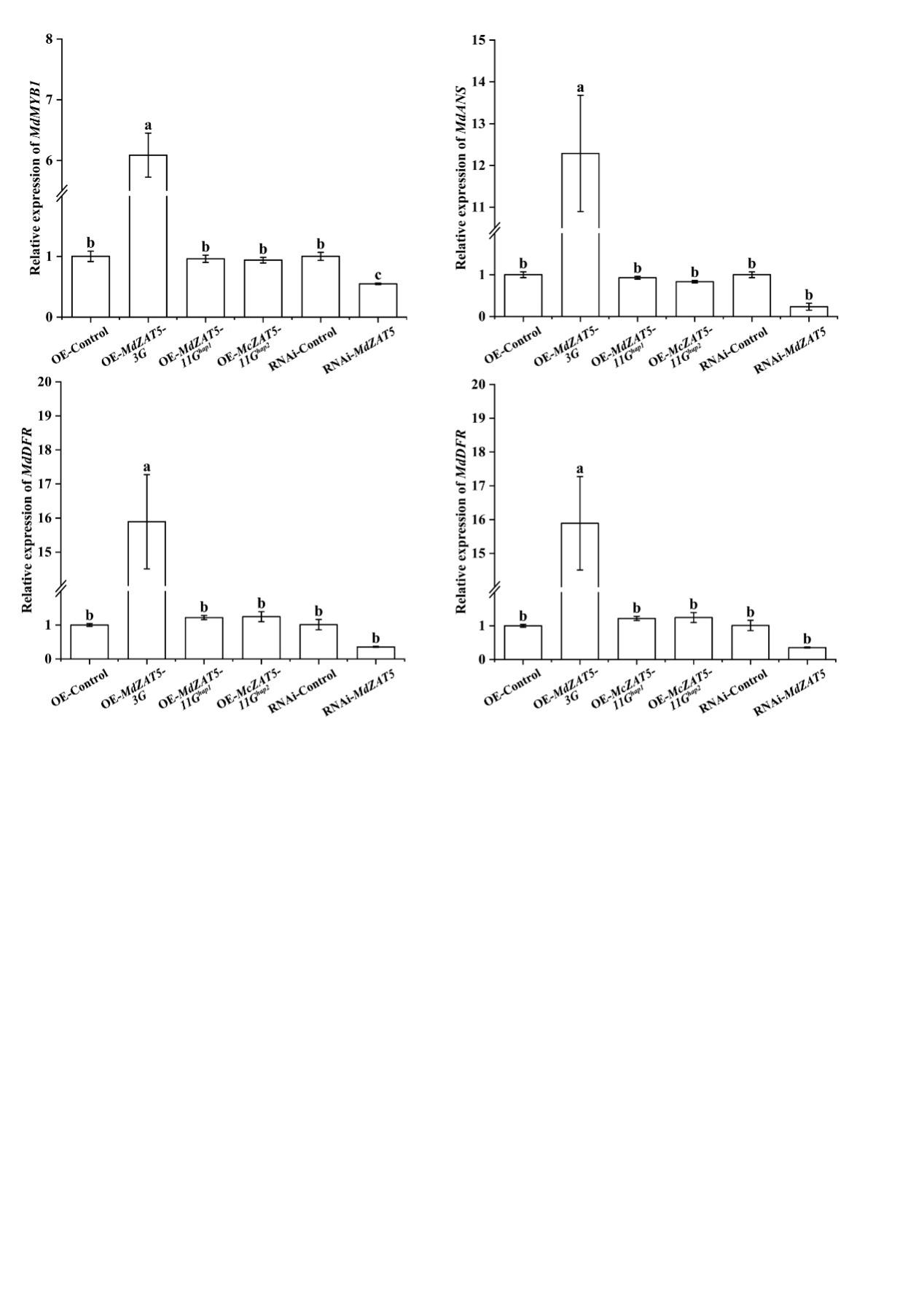
**Figure S7.** Relative expression levels of *MdMYB1* and the anthocyanin biosynthesis genes *MdDFRS*, *MdANS* and *MdUFGT* in transgenic apple calli as detected by RT-qPCR. The RT-qPCR analysis were performed with three biological replicates. Error bars indicate the standard error of the mean ± SE of three replicate measurements. Different letters above the bars indicate significantly different values (*P* < 0.05), calculated using one-way analysis of variance (ANOVA) followed by a Tukey’s multiple range test

**
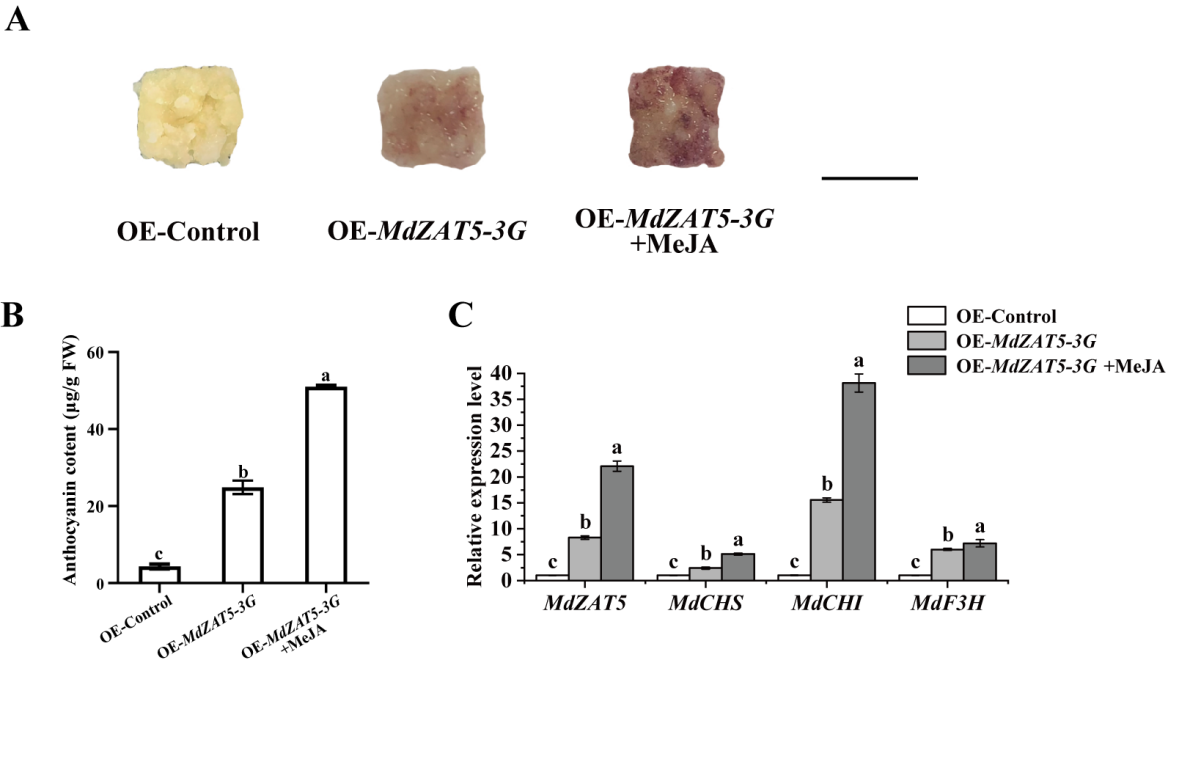
**

**Figure S8.** Effects of MeJA on anthocyanin accumulation in OE-MdZAT5-3G calli.(A) OE-MdZAT5-3G calli were treated with MeJA. MeJA significantly promoted anthocyanin accumulation. (B) **Anthocyanin content in transgenic apple calli shown in (A). (C) Relative expression levels of *MdZAT5-3G* and anthocyanin biosynthesis genes *MdCHS*, *MdCHI* and *MdF3H* in transgenic calli determined by RT-qPCR. Expression levels were normalized to the empty vector or wild type (set to 1). All RT-qPCR and anthocyanin assays were performed with three biological replicates. Error bars represent the mean ± standard error (SE) of three replicates. Different letters above bars indicate statistically significant differences (*P*** **< 0.05), determined by one-way ANOVA followed by Tukey’s multiple range test.**

**
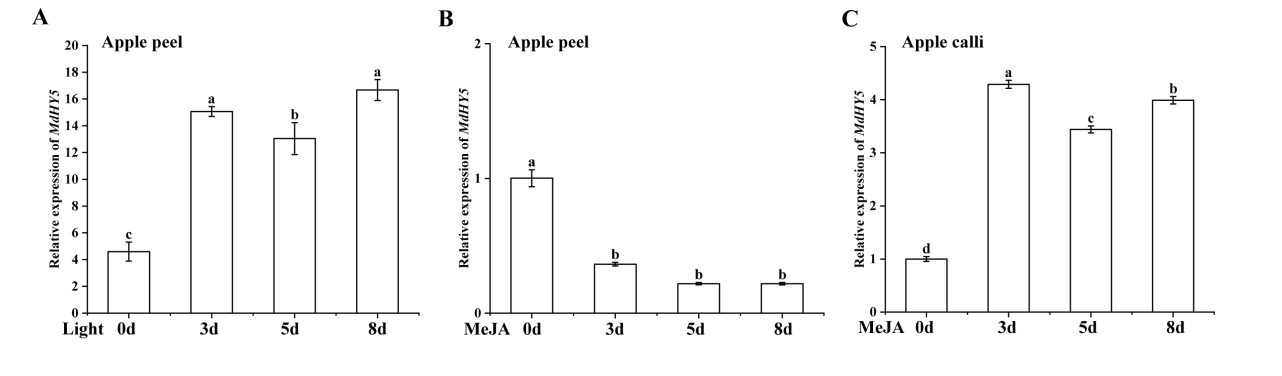
**

**Figure S9.** Relative expression levels of *MdHY5* in apple peel following light and MeJA treatment and apple calli following MeJA treatment. The RT-qPCR analysis were performed with three biological replicates. Error bars indicate the standard error of the mean ± SE of three replicate measurements. Different letters above the bars indicate significantly different values (*P* < 0.05), calculated using one-way analysis of variance (ANOVA) followed by a Tukey’s multiple range test.


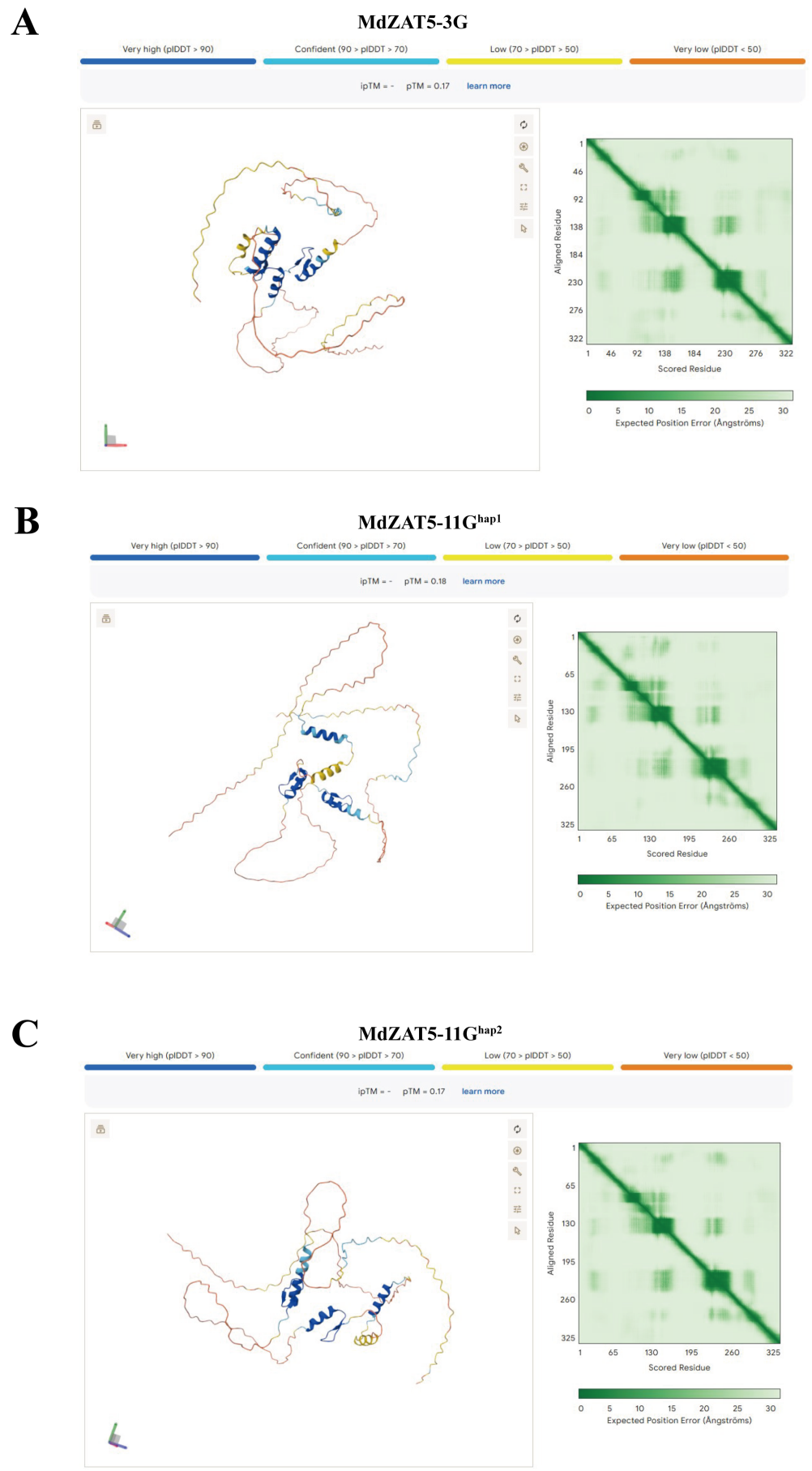


**Figure S10.** Predicted protein structures of MdZAT5-3G, MdZAT5-11G^hap1^, MdZAT5-11G^hap2^ generated using AlphaFold. The pLDDT values indicate the local structural accuracy of each chain, with higher scores reflecting greater confidence in the predicted regions.
